# A Closed‐Loop Framework for Inverse Design: Dynamic Training and Intelligent Optimization for Heterostructured Materials

**DOI:** 10.1002/advs.76524

**Published:** 2026-07-20

**Authors:** Zhiyan Zhong, Xueru Zheng, Xiao Zhou, Zhengheng Tao, Wan Han, Jingyi Pan, Dian Wu, Ning Gao, Lei Liu, Zhongyang Wang, Fanchao Meng, Tongxiang Fan

**Affiliations:** ^1^ State Key Lab of Metal Matrix Composites School of Material Science and Engineering Shanghai Jiao Tong University Shanghai P. R. China; ^2^ Shanghai Innovation Institute Shanghai P. R. China; ^3^ Institute of Medical Robotics Shanghai Jiao Tong University Shanghai P. R. China; ^4^ School of Mathematical Sciences Peking University Beijing P. R. China; ^5^ Institute of Advanced Studies in Precision Materials Yantai University Yantai Shandong P. R. China

**Keywords:** heterostructured metal matrix composites, inverse design, machine learning, mechanical properties, microstructure

## Abstract

For decades, heterostructured materials have attracted great research interest because of their remarkable mechanical and physical properties. Nevertheless, exploring the immense design space to address the intrinsic trade‐off between strength and toughness through trial‐and‐error experiments and simulation approaches entails considerable temporal and economic expenditures. Here, we propose a comprehensive end‐to‐end scientific machine learning framework integrating a deep learning model (Back‐Propagation Neural Network with Continual Learning, BPNN‐CL) for forward prediction and an optimization algorithm (NSGA‐II with Partition Monitoring and Chaotic Perturbation, NSGA‐II‐PMCP) for inverse optimization, and demonstrate its effectiveness using metal matrix composites as representative heterostructured materials. The forward prediction using BPNN‐CL achieves errors within 10% for most experimental results, and decreases the Mean Absolute Percentage Error of elastic modulus’ testing dataset more than 14.9% compared with six conventional machine learning methods. In contrast to conventional approaches (e.g. NSGA‐II, Bayesian optimization, et al.), NSGA‐II‐PMCP enables inverse design with enhanced generalization and high efficiency, and maintaining broad applicability across diverse material systems and properties. This work establishes a unified platform for heterostructured material design that, when integrated with emerging cutting‐edge manufacturing techniques such as 3D printing, offers a readily extensible approach to accelerate the development of diverse advanced heterostructured materials.

## Graphical abstract

To accelerate the inverse design of heterostructured metal matrix composites, a closed‐loop scientific machine learning framework integrates continual learning prediction with NSGA‐II‐PMCP optimization. The framework maps microstructural descriptors to strength, toughness, and modulus, expands high‐quality Pareto solutions, and guides experimentally validated SiCp/Al composites with customized mechanical performance.

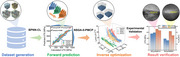

## 1 Introduction

Heterostructured (HS) materials, ubiquitously existing in nature [1], exhibit exceptional mechanical properties [2, 3, 4] and broad applications in engineered structures [5, 6]. Among these, heterostructured metal matrix composites (HSMMCs) are promising for aerospace [7, 8] and automotive [9] applications due to low density [10, 11], high specific strength [12, 13], and high specific modulus [14, 15]. Although these exceptional properties make HSMMCs attractive for diverse applications, their widespread adoptions are still hindered by the inherent strength‐toughness trade‐off [16, 17, 18]. Overcoming this challenge requires the establish of precise structure‐property relationships [19, 20], yet vast design space renders trial‐and‐error approaches impractical [21, 22, 23]. Although analytical models enable rapid analysis, they offer only oversimplified physical insights (e.g., Eshelby Inclusion Model [24, 25], Griffith Fracture Criterion [26]), being far from real complicated cases. Meanwhile, high‐fidelity numerical methods (e.g., phase‐field modeling [27], finite‐element analysis (FEA) [28]) can capture detailed and critical physics but come at prohibitively high computational costs in complex material combinations. Fortunately, advances in machine learning (ML) offer a feasible venue to address these challenges [29, 30, 31].

More recently, ML methods have rapidly gained attention due to its proficiency in uncovering complex relationships between input descriptors and target properties [32, 33, 34, 35]. Moreover, instead of merely mapping the relation from structural parameters to mechanical properties, ML models combine with optimization algorithms can establish mappings from desired mechanical properties back to structural parameters, enabling so‐called inverse design [36, 37, 38]. In this regard, the current ML‐assisted inverse design framework, which comprises two key components: (i) forward prediction [13, 39, 40, 41]: structure → properties; and (ii) inverse optimization [42, 43]: the exploration for mechanical properties → optimal structures, which represents a promising alternative for inverse design.

Recent advances in forward prediction models bridge microstructures to properties [42, 44]. However, current ML models show limitations for HSMMCs. On the one hand, although conventional and static ML methods excel in HS thermal conductivity modeling [45], surface roughness prediction [46], and image recognition [47], they still lack dynamic training capability and thus limit scalability and adaptation to evolving datasets [48, 49]. For example, Nosonovsky et al. developed models to predict wear rate and friction in aluminum‐graphene composites [50], but modifications to material constituents (e.g., matrix replacement) necessitate model retraining. On the other hand, while other algorithms like Convolutional Neural Networks (CNNs) [51] and long short‐term memory (LSTM)) [52, 53] are widely applied in process parameter prediction [54], stress‐strain curve modeling [55], and images segmentation [56], they remain fundamentally vulnerable to catastrophic forgetting, which refers to as the tendency to rapidly lose previously learned knowledge when models are adapted to new data or tasks [57]. For instance, despite Gao et al. proposed a powerful nonlinear CNNs‐LSTM model for multiaxial fatigue life prediction in metallic materials [58], these architectures fail to mitigate catastrophic forgetting when sequentially trained on large‐scale datasets. Moreover, transfer learning [59, 60, 61], a widely used dynamic training paradigm, has important limitations because its pretrained feature extractors remain largely fixed after transfer, restricting deeper task‐specific adaptation. Thus, a forward prediction framework integrating dynamic training and catastrophic forgetting resistance could transcend these limitations.

In addition, inverse optimization uses the forward‐prediction model as a surrogate model and applies multi‐objective optimization to explore optimal microstructural features [42, 62]. For instance, Genetic Algorithm (GA) simulates natural evolution [63, 64, 65] and effectively explores vast solution spaces, making GAs ideal for complex design problems [66, 67, 68, 69]. Nonetheless, local optima susceptibility and static model constraints are two critical obstacles. When addressing high‐dimensional problems, there remain local optimization limitation. For instance, our previous implementation of random forest regression (RFR) with NSGA‐II (RFR‐NSGA‐II) for microstructure exploration is still unsatisfied for global optimization [70]. It is worth noting that analogous ML‐driven microstructure design strategies have been demonstrated in heterostructured materials systems. For instance, Fukatsu et al. combined GANs with FEA simulations and Bayesian optimization (BO) to maximize the strength–ductility balance of dual‐phase steels, achieving microstructural designs that outperformed real steels [71, 72, 73]. However, such an integrated generative‐optimization framework has yet to be established for HSMMCs, where particle reinforcement geometry and interface characteristics pose substantially greater complexity. Therefore, addressing GA's local optima while systematically integrating the aforementioned forward models is critical for achieving optimal Pareto solutions.

Herein, we proposed an innovative, end‐to‐end inverse design framework featuring two key advances: (i) Forward prediction: We integrate a BPNN with continual learning (CL), which is capable of dynamic training while mitigating catastrophic forgetting, to develop a BPNN‐CL model; (ii)Inverse optimization: We developed an NSGA‐II‐PMCP algorithm by integrating partition monitoring (PM) and chaotic perturbation (CP) into the NSGA‐II, using the BPNN‐CL as a surrogate to enable global optimization. The framework is validated on particle‐reinforced aluminum (Al) matrix composites (PRAMCs), a representative heterostructured system widely deployed materials. Our framework demonstrates significant superiority across three critical aspects: (i) Properties prediction: the R^2^ of ultimate tensile strength exceeds 0.98 and toughness surpasses 0.94. In addition, the forward prediction using BPNN‐CL achieves errors within 10% for most examined experimental data, and decreases the Mean Absolute Percentage Error (MAPE) of elastic modulus’ testing dataset more than 14.9% compared with six conventional machine learning methods; (ii) Generalization: The hypervolume (HV) of NSGA‐II‐PMCP shows a 68.95% increase over standard NSGA‐II and Pareto solution set significantly outperforms Bayesian optimization; (iii) Optimization time: NSGA‐II‐PMCP simultaneously achieves superior with reduced computational time. We anticipate that this framework will pave the way for next‐generation HS materials with tailored properties and functionalities, particularly under data‐limited conditions.

## 2 Results and Discussion

### 2.1 Framework of Inverse Design Heterostructured Materials

**FIGURE 1:**
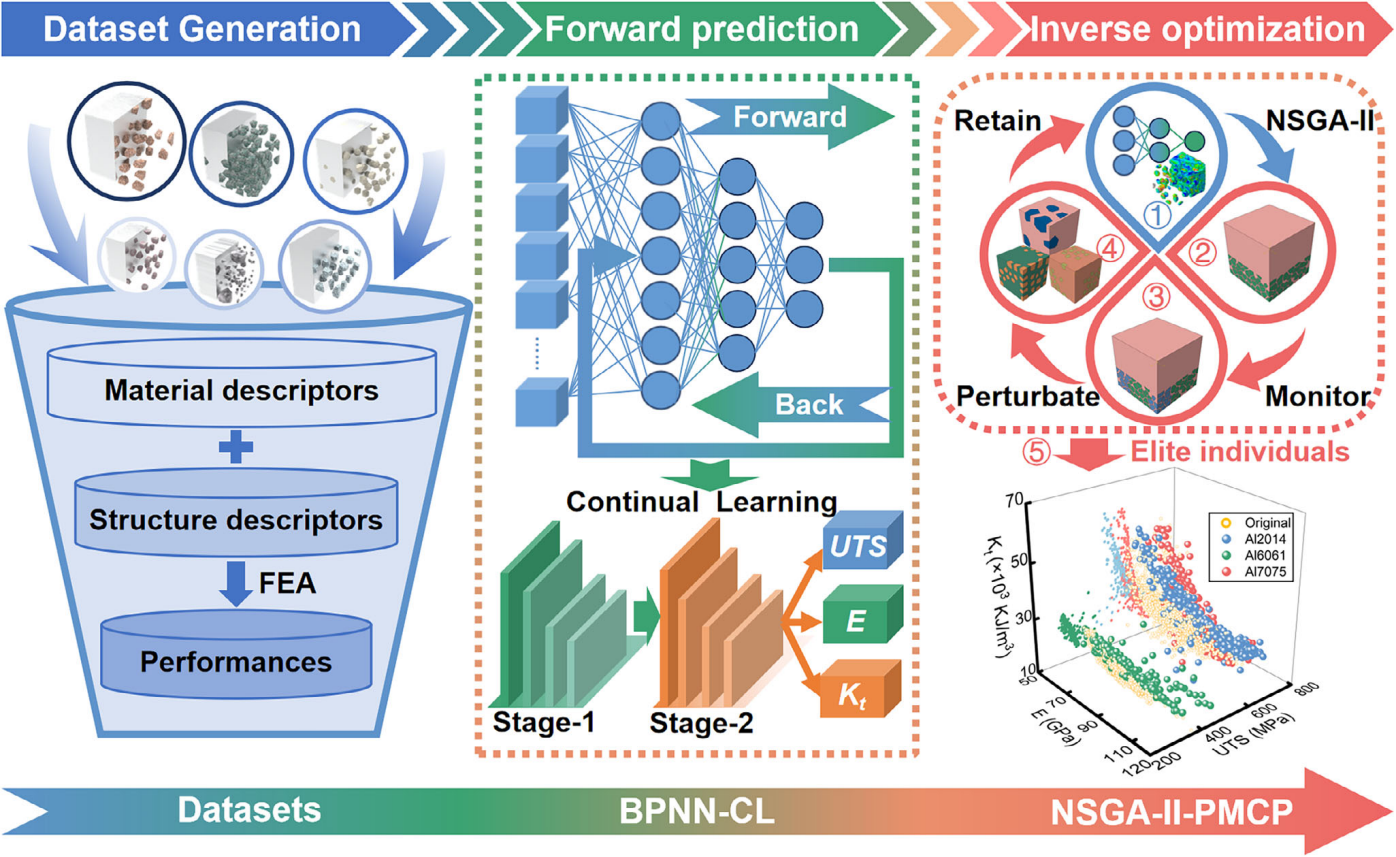
Overview of the ML‐based forward and inverse design. 2, 718 heterostructured metal matrix composites (HSMMCs) are generated from material and structure descriptors. Finite element analysis (FEA) under uniaxial tension is performed to obtain properties including strength, toughness, and elastic modulus. (A) forward prediction model (BPNN‐CL) is established by integrating a BPNN with two stage continual learnings (CL), thereby enabling precise mapping from descriptors to mechanical properties (*UTS*, *K_t,_* and *E*). For inverse optimization, we introduce the NSGA‐II‐PMCP, which augments NSGA‐II with partition monitoring (PM), chaotic perturbations (CP), and retains elite individuals. BPNN‐CL serves as the surrogate model for NSGA‐II‐PMCP, enabling the efficient inverse design of microstructures with optimal mechanical properties.

We implement an inverse design framework (Figure 1) comprising dataset generation, forward prediction, and inverse optimization. For dataset generation, material descriptors (describing matrix) and structural descriptors (describing interfaces and reinforcements) are paired with mechanical properties including ultimate tensile strength (*UTS*), toughness (*K_t_*), and elastic modulus (*E*) to form the dataset. The forward prediction, BPNN‐CL, is built by pretraining a back‐propagation neural network (BPNN) on the initial dataset followed by two sequential continual‐learning stages (Stage‐1 and Stage‐2) to assimilate new data while mitigating catastrophic forgetting. Evaluated across multiple PRAMCs, BPNN‐CL shows superior predictive accuracy compared with mainstream machine learning baselines and agrees with reported experimental values. For inverse optimization, NSGA‐II is augmented with chaotic perturbation (CP), partition monitoring (PM), and elite retention to produce NSGA‐II‐PMCP workflow, while BPNN‐CL serves as a real‐time surrogate to accelerate objective evaluation and helping escape local optima. NSGA‐II‐PMCP achieves broader Pareto coverage, faster convergence, and higher hypervolume than Bayesian optimization and standard NSGA‐II. The optimized elites are finally validated by FEA and experiments.

### 2.2 HSMMCs Microstructure and Dataset

We develop a dataset of a total 2, 718 HSMMC samples with parameterized structures and material descriptors. It should be noted that the experimental engineering stress–strain curves and the simulated microstructural responses are not fully equivalent over the entire deformation range. Experimental tensile results are inevitably affected by specimen‐level necking, strain localization, and final fracture, whereas the finite‐element simulations represent the effective response of a representative microstructure under idealized uniaxial loading. In addition, the fracture mode of PRAMCs may vary from ductile to mixed or more localized fracture depending on particle volume fraction, particle distribution, reinforcement architecture, and interfacial condition. Principle S3 analyzed the influence of specimen‐level necking and fracture. Figure S1 illustrates the representative reinforcement configurations and Figure S2 presents the HSMMCs dataset and microstructural features, including *UTS*–*K_t_*–*E* scatter plots, correlation analysis, and descriptor distributions. It is essential to define the complex logical relationships among these numerous parameters as the mechanical properties of HSMMCs stem not from any individual factor, but from the nonlinear interactions between the material properties and the spatial characteristics of the matrix, interface products (Al_4_C_3,_ Mg_2_Si, and Al_4_Si_3_), and reinforcements (SiC). Specifically, material descriptors are list in Table 1. Figure S3–S6 shows the multi‐configuration fracture analysis of HSMMCs based on various aluminum alloy matrices, including Al2014, Al2024, Al6061, and Al7075, which are representative aluminum alloys widely used as matrix systems in PRAMCs.

**TABLE 1:** Main symbol explanations in the article.

Parameters	Symbols	Parameters	Symbols	Parameters	Symbols
Fracture strain	D1	Interface volume	D7	Reinforcement diameter	D13
Matrix elastic modulus	D2	Thickness of interface	D8	Reinforcement volume fraction	D14
Yield strength(*A*)	D3	Al_4_C_3_ ratio*	D9	Reinforcement diameter variance	D15
Hardening modulus(*B*)	D4	Mg_2_Si ratio*	D10	Ultimate Tensile Strength (*UTS*)	P1
Hardening exponent(*n*)	D5	Al_4_Si_3_ ratio*	D11	Toughness (*K_t_*)	P2
Rate hardening parameter(*C*)	D6	Reinforcements configurations	D12	Elastic modulus (*E*)	P3

*The ratio refers to the proportion of an individual interfacial product relative to the total interfacial products.

### 2.3 Descriptors Screening

**FIGURE 2:**
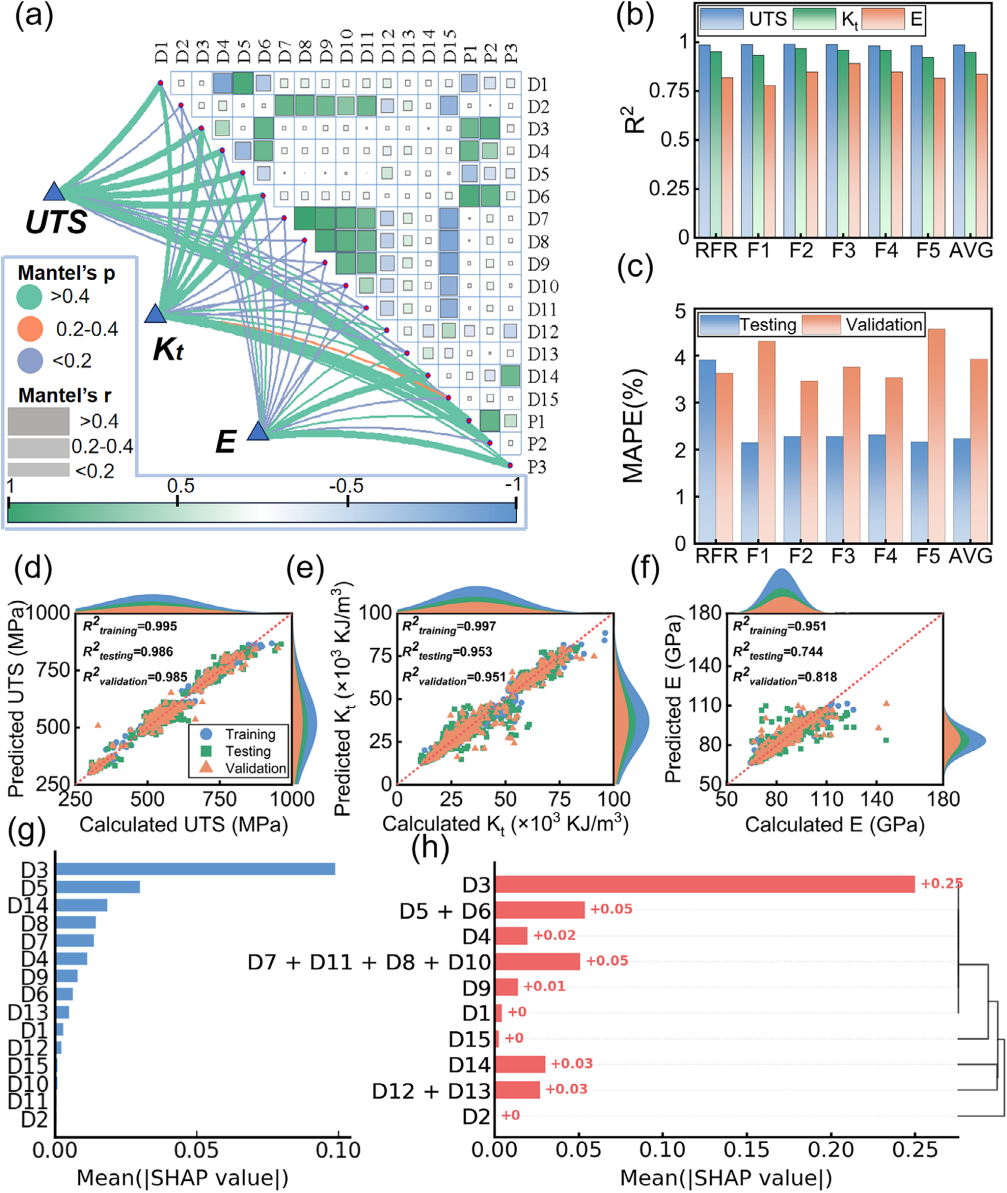
Descriptors screenings. (a) Mantel tests between properties (*UTS*, *K_t_* and *E*) and descriptors (D1‐D15). Edge color and width indicate Mantel's *p* and *r* values, respectively, where lower *p*‐values and higher *r*‐values indicate stronger and more significant matrix correlations. The heatmap color gradient denotes the Pearson correlation coefficients among descriptors (listed in Table 1). (b) R^2^ values for *UTS*, *K_t_*, and *E* predictions on the validation set using the RFR model and five‐fold cross‐validation. (c) MAPE values for testing and validation sets obtained via RFR with five‐fold cross‐validation. Comparison of predicted versus calculated values of *UTS* (d), *K_t_* (e), and *E* (f) across training, testing, and validation sets. Histograms on the top and right illustrate that all three sets are well distributed across the entire property range. (g) The descriptors’ SHAP feature‐importance ranking. (h) SHAP clustering analysis of descriptor contributions, identifying groups of descriptors that exhibit similar effects on model predictions and highlighting dominant features.

We initially employ the Mantel test to identify the dominant drivers among descriptors for the three target properties (i.e., *UTS, K_t_, E*). In Figure 2a, Mantel tests show the relationship between properties and descriptors. Edge color and width correspond to Mantel's *p* and *r* values, respectively, where lower *p*‐values and higher *r*‐values indicate stronger and more significant matrix correlations. The heatmap color gradient denotes the Pearson correlation coefficients among descriptors. Combined with Figure S8, Mantel tests reveal distinct control mechanisms: *UTS* and *K_t_* exhibit a strong negative correlation with matrix fracture strain and *n* (Pearson's r < −0.5) but a positive correlation with *A* and *B* (r > 0.5). In contrast, *E* is predominantly governed by the reinforcement volume fraction (r > 0.5).

We further applied RFR combined with SHapley Additive ExPlanations (SHAP) to identify key descriptors. Figure 2b reports the R^2^ values for the validation set using the RFR model with five‐fold cross‐validation and Figure 2c shows the Mean Absolute Percentage Error (MAPE) for testing and validation sets. The consistently high R^2^ and low MAPE indicate the RFR is robust and not overfitting. Figure 2d–f compare calculated haod versus predicted *UTS*, *K_t_* and *E* using scatter plots: the red dashed diagonal denotes perfect agreement, proximity to this line indicates prediction accuracy, and the marginal shaded curves show the normal distributions of training, testing and validation. *UTS* exhibits the highest R^2^, followed by *K_t_*. Specifically, the R^2^ of the training set in exceed 0.95, and the Root Mean Squared Error (RMSE), Mean Absolute Error (MAE), and MAPE of five‐fold cross‐validation below 0.05 (Figure S7). For the SHAP analysis, Figure 2g presents the feature‐importance ranking of the descriptors. Yield strength A (D3) and hardening exponent n (D5) show the largest contributions among the matrix‐related constitutive descriptors, confirming that the strength and hardening differences are explicitly encoded by D3–D6 and captured by the trained model. In contrast, the contributions from D2 and D11 are negligible. Figure 2h is the SHAP clustering of descriptor contributions, identifying groups of descriptors that exhibit similar effects on model predictions. (Figure S8 for details). Quantitatively, contributions from matrix elastic modulus and Al_4_Si_3_ ratio are below 0.1% (Table S3), which justifies their exclusion.

### 2.4 BPNN‐CL for Forward Prediction

**FIGURE 3:**
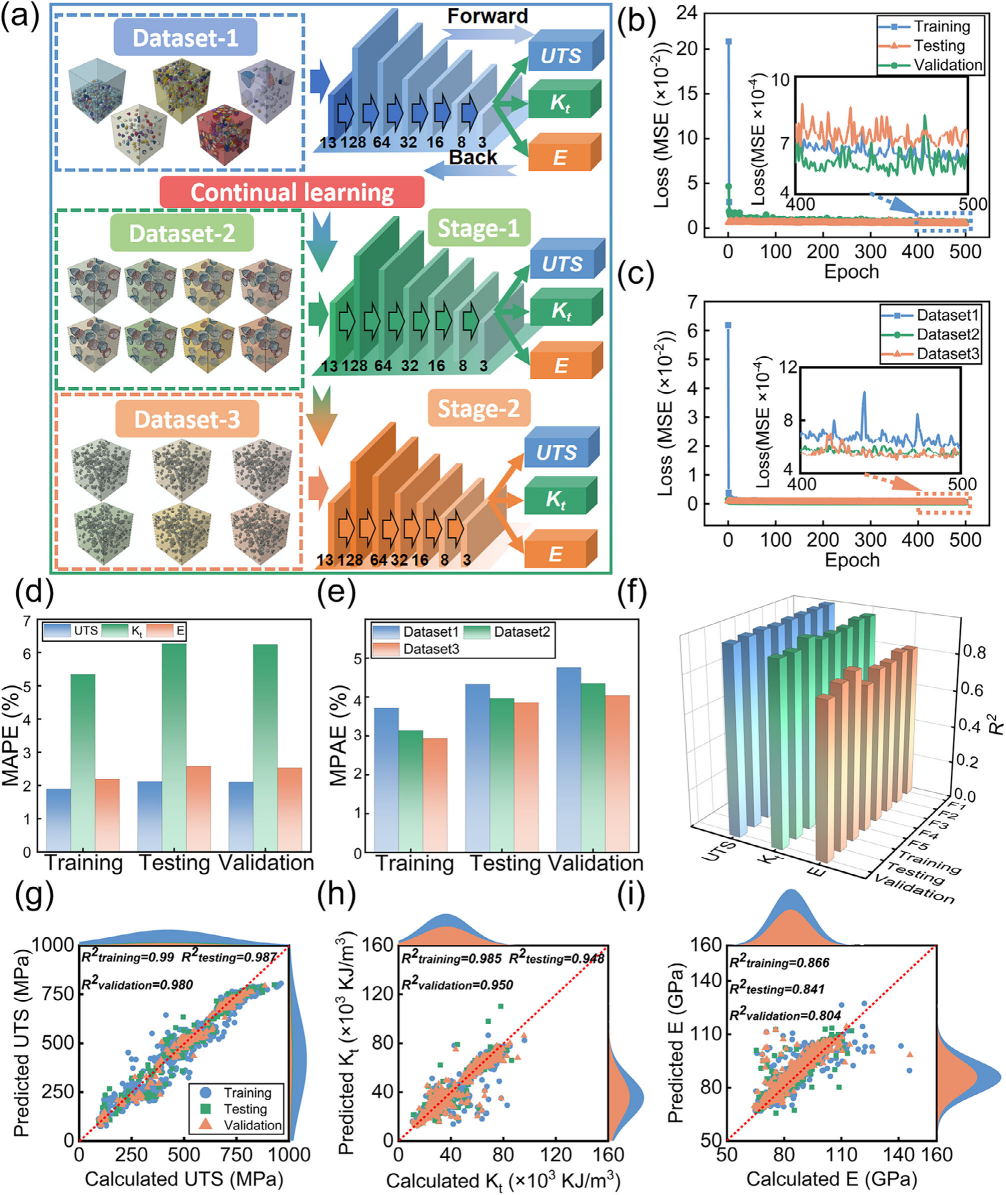
Properties of the BPNN‐CL model in predicting. (a) Schematic of the BPNN‐CL architecture. The workflow begins with pretraining a BPNN on Dataset 1, followed by two continual learning stages using Datasets 2 and 3. The BPNN takes 13 input features (descriptors), includes five hidden layers with 128, 64, 32, 16, and 8 neurons, and outputs 3 properties (*UTS*, *K_t_*, and *E*). The continual learning stages (Stage‐1 and Stage‐2) are built upon the pretrained BPNN, maintaining the same input features, and output dimensions. (b) The convergence behavior of the training, testing, and validation sets of Dataset 1 within the pre‐trained BPNN. (c) The overall convergence properties across Datasets 1–3 during the pre‐trained BPNN, Stage‐1, and Stage‐2 continual learning processes. The loss function is defined as the total error of *UTS*, *K_t_*, and *E*, reflecting the overall predictive properties of the model. (d) The MAPE of *UTS*, *K_t_*, and *E* across the training, testing, and validation sets under the BPNN model. (e) MAPE values of *UTS*, *K_t_*, and *E* predicted by the BPNN‐CL. (f) R^2^ of *UTS*, *K_t_*, and *E* predicted by the BPNN‐CL. Calculated versus predicted values of *UTS* (g), *K_t_* (h), and *E* (i) obtained by the BPNN‐CL model across training, testing, and validation sets. Histograms on the top and right illustrate that all three sets are well distributed across the entire property range.

We then introduce a BPNN with continual learning (BPNN‐CL) for properties prediction. By integrating continual learning, the BPNN‐CL model continually incorporates new PRAMCs data while retaining prior knowledge, ensuring resistance to forgetting and continuous model improvement. As illustrated in Figure 3a, the BPNN is first pre‐trained using Dataset 1 (a high‐properties dataset without interfacial products). In particular, the BPNN has 13 input features (descriptors) and includes five hidden layers. After training, the pre‐trained model can output 3 target mechanical properties (*UTS*, *K_t_*, and *E*). Subsequently, stage‐1 and stage‐2 continual learning are conducted using Dataset 2 (a random configuration dataset without interfacial products) and Dataset 3 (a random‐configuration dataset with interfacial products) respectively. All continual learnings maintain the same input features, hidden‐layer structure, and output dimensions with BPNN.

Figure 3b presents the convergence curves of the pre‐trained BPNN for training, testing, and validation sets over 0–500 epochs (main panel) with a 400–500 epoch zoom inset, showing that the testing set converges fastest while the validation set converges more slowly. Figure 3c compares convergence across Datasets 1–3 for pretraining, Stage 1 and Stage 2 continual learning, revealing that Stage 2 converges about 83.3% faster than BPNN pretraining. Additionally, Figure 3d presents the MAPE of *UTS*, *K_t_*, and *E* across the training, testing, and validation sets for the pre‐trained BPNN, while Figure 3e shows the corresponding overall MAPE after full BPNN‐CL training. Figure 3f shows R^2^ for the well‐trained BPNN‐CL on training, testing, and validation sets, as well as the results from five‐fold cross‐validation (F1–F5); the close agreement between these values indicates reproducible properties and no overfitting. Figure 3g–i compare calculated versus predicted *UTS*, *K_t_* and *E* using scatter plots: the red dashed diagonal denotes perfect agreement, proximity to this line indicates prediction accuracy, and the marginal shaded curves show the normal distributions of training, testing and validation. *UTS* exhibits the highest R^2^, followed by *K_t_*; although *E* has a lower R^2^ overall, the testing R^2^ for *E* from BPNN‐CL is 11.53% higher than that from RFR and its distribution remains stable, indicating superior generalization of the BPNN‐CL model.

This work conducts a comparison with seven famous machine learnings in Table 2 (hyperparameters are detailed in Table S4) including Linear Regression (LR), Decision Tree (DT), RFR, Support Vector Machine (SVM), Gaussian Process (GP), and k‐Nearest Neighbors (KNN), Neural Network (NN). Although SVM provides a competitive baseline, BPNN‐CL reduces the testing and validation MAPE for *E* by approximately 14.9% and 10.3%, respectively. Compared with the best properties method in our previous work (RFR) [70], BPNN‐CL improved *E* prediction by 3.83% and 7.2% in testing and validation R^2^, respectively. Additionally, BPNN‐CL outperforms all other models across nearly every metric: it reduces the MAPE testing sets for *E* by ≥14.9% relative to LR, DT, SVM, KNN, and NN, and by a remarkable ≥72.6% relative to GP in the testing dataset. These results demonstrate that the BPNN‐CL not only exhibits excellent predictive accuracy but also outperforms substantially mainstream conventional machine learning methods. To compare computational time and memory complexity on the same footing, all models were evaluated on identical data and hardware using per‐sample inference time, memory footprint, and parameter count.

**TABLE 2:** Comparison between traditional machine learning methods and this work.

Method	Datasets	*UTS*		*K_t_*		*E*	
		MAPE	R^2^	MAPE	R^2^	MAPE	R^2^
LR	Testing	3.30%	0.976	8.98%	0.927	3.91%	0.674
	Validation	3.49%	0.971	9.19%	0.936	3.76%	0.743
DT	Testing	2.89%	0.969	8.24%	0.920	3.42%	0.690
	Validation	2.74%	0.974	8.35%	0.928	3.49%	0.614
RFR	Testing	2.18%	0.982	7.18%	0.947	2.90%	0.810
	Validation	2.23%	0.981	7.09%	0.947	2.82%	0.750
SVM	Testing	2.92%	0.981	7.43%	0.936	3.02%	0.774
	Validation	3.02%	0.981	7.28%	0.947	2.82%	0.840
GP	Testing	29.69%	0.017	50.48%	0.017	9.38%	0.016
	Validation	30.04%	0.020	50.44%	0.022	9.45%	0.026
KNN	Testing	2.42%	0.981	7.64%	0.937	3.45%	0.725
	Validation	2.39%	0.981	7.29%	0.948	3.32%	0.781
NN (5‐Layer)	Testing	2.51%	0.982	7.28%	0.942	3.90%	0.7416
	Validation	2.55%	0.983	7.14%	0.955	2.79%	0.806
This work	Testing	2.12%	0.987	6.27%	0.948	2.57%	0.841
	Validation	2.10%	0.980	6.25%	0.950	2.53%	0.804

As shown in Table S7, BPNN‐CL has 12 819 trainable parameters, slightly fewer than the matched five‐layer NN baseline with 13 983 parameters, indicating that the continual learning mechanism does not increase model size. Its inference time and memory footprint are 2.605 ms and 18.390 KB, respectively, which remain within a practical millisecond‐level range and are lower than those of Random Forest in time cost and Gaussian Process in memory cost. Although BPNN‐CL is slower than the one‐shot five‐layer NN because of staged weight management and weight composition during inference, this overhead is accompanied by substantial accuracy improvements, particularly a great gain for elastic modulus. Thus, BPNN‐CL achieves a meaningful trade‐off between computational cost and predictive performance rather than introducing redundant complexity.

**FIGURE 4:**
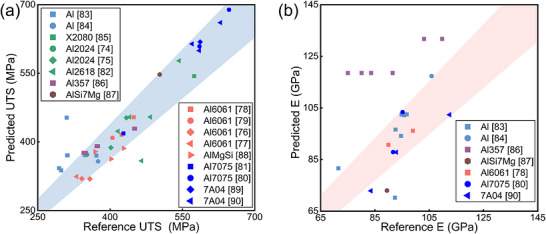
Comparison of the predicted and reference SiC‐reinforced Al matrix composites for different matrices and processes. Panels show the *UTS* (a) and *E* (b) comparisons, respectively, where the shaded regions indicate ±10% error intervals (light blue for *UTS* and light red for *E*).

To further validate BPNN‐CL against experiments, literature‐reported *UTS* values for SiC‐reinforced Al matrix composites are compared with model predictions in Figure 4 (see Table S8 for details). It should be noted that literature‐reported data comes from various fabrication/processing, which could definitely affect the final mechanical properties. To account for processing‐induced variability, we extract the stress–strain curves of each alloy matrix under different fabrication conditions from the literature and recalculated the corresponding descriptors (D1–D15) for each specific processing route. These processing‐dependent descriptors are then used as inputs to the trained BPNN‐CL model, enabling the model to predict properties for SiCp/Al composites under matched processing conditions. The predicted values are subsequently compared with the experimental results to ensure that variations in processing are properly reflected in both the input representation and performance evaluation. The comparison covers two groups: (1) variants of alloys of the same types present in the original training data—Al2024 [74, 75], Al6061 [76, 77, 78, 79], Al7075 [80, 81], Al2618 [82]—but processed under different process conditions. And (2) alloys that are not included in the training set, namely pure Al [83, 84], X2080 [85], A357 [86], AlSi7Mg [87], AlMgSi [88], 7A04 [89, 90]. Figure 4a compares the BPNN‐CL's *UTS* predictions with literature values, the light shaded band denotes a ±10% error range, and points near the diagonal indicate smaller discrepancies. Predicted *UTS* agrees closely with experiments, with most points within ±10%. Notably, the model interpolates well within the trained strength range (300–700 MPa) demonstrating robust interpolation within the known design space. Moreover, several matrices that are not included in the training set are also predicted with high accuracy, indicating generalizability across unseen PRAMCs. However, the larger deviations occur in materials such as pure Al, whose *UTS* (100‐250 MPa) falls entirely outside the trained *UTS* range. This confirms that the model performs reliably within its training domain but struggle to extrapolate accurately beyond the represented property range. Similarly, Figure 4b compares the BPNN‐CL's *E* predictions with the corresponding literature data (the light blue shaded region indicates ±10% error range). Specifically, significant outliers correspond exclusively to matrices, which is not included in the training set (pure Al, A357, AlSi7Mg, and 7A04), whereas Al6061 and Al7075 display excellent agreement with experiments. Herein, the predictions and experiments comparison substantiate the BPNN‐CL applicability for forward design, while also highlighting the continued importance of representative training coverage when extending predictions to novel matrix compositions.

Overall, BPNN‐CL outperforms conventional static models by continually updating mappings while preserving prior knowledge. A replay buffer interleaves new and old samples to maintain key feature representations without full retraining, and an explicit regularization term limits weight drift so adaptations to new reinforcement architectures do not erode existing correlations. Its gradient‐based training captures complex nonlinear interactions more faithfully than piecewise methods such as RFR. Together, these mechanisms sustain high R^2^, reduce MAE and MAPE, and enable much faster incremental updates, establishing BPNN‐CL as a robust, scalable surrogate for evolving PRAMC design.

### 2.5 NSGA‐II‐PMCP for Inverse Optimization

Next, we focus on the inverse design approach from properties to structures. As discussed above, the design space for PRAMCs is enormous and highly complex. Even though the BPNN‐CL model achieves high predictive accuracy, it alone can't resolve the inverse design problem, since the complicated microstructures may correspond to similar properties outcomes, thereby creating an uncertain search space. Hence, successful inverse design requires not only predictive accuracy but also a dedicated optimization framework. To address this challenge, we use the pre‐trained BPNN‐CL model to predict *UTS*, *K_t_*, and NSGA‐II‐PMCP as optimizer to map properties to microstructures.

**FIGURE 5:**
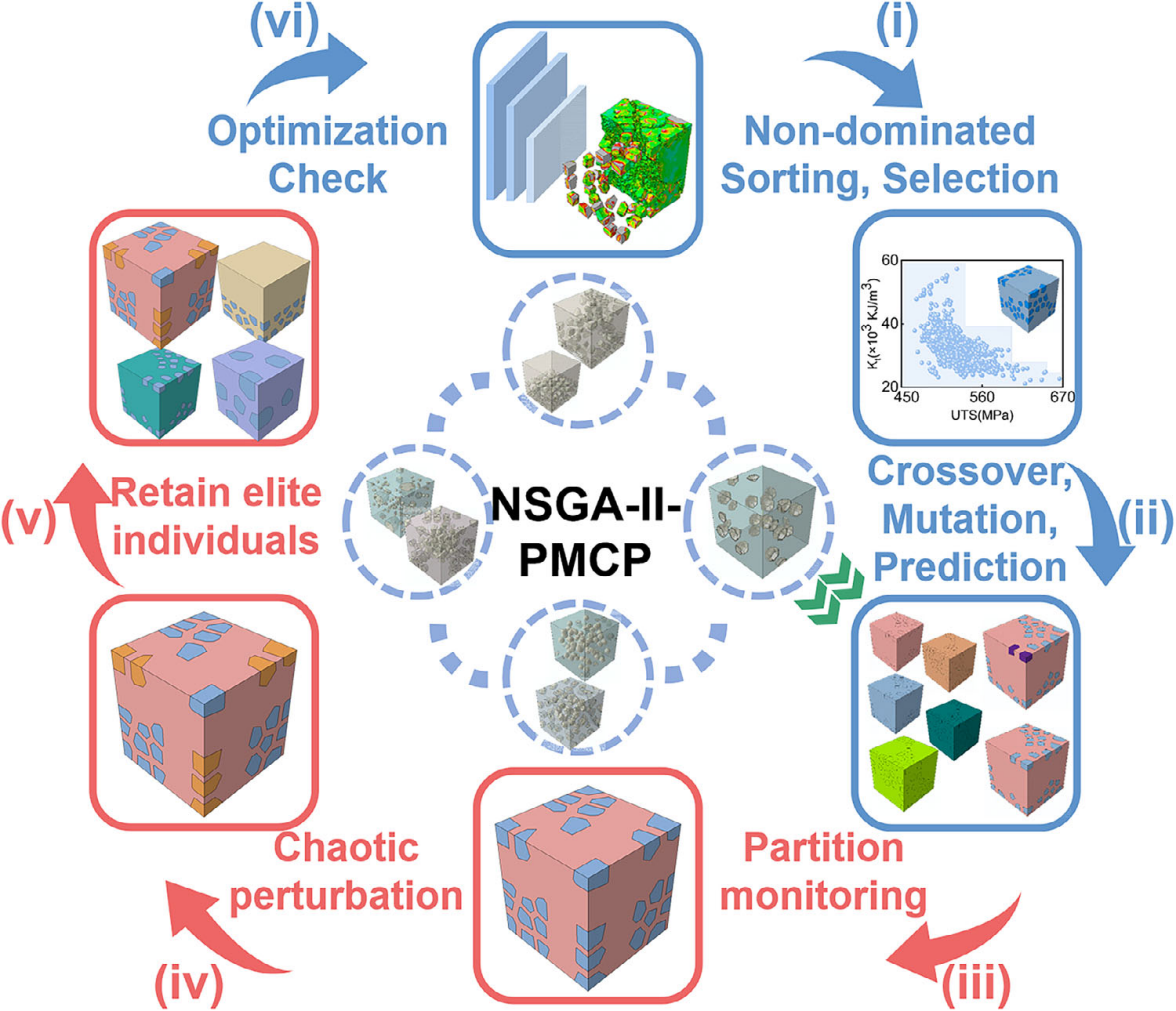
Schematic of the NSGA‑II‑PMCP inverse optimization. First, NSGA‐II undergoes non‐dominated sorting and parent microstructures are chosen for crossover and mutation. Partition monitoring detects regions of the front with sharp properties changes per 20 generations and focuses the search on these local subdomains. When the population shows reduced diversity, chaotic perturbations are introduced to restore exploration capability. Elite solutions are carried forward across generations to retain the best‐performing designs. This iterative cycle continues until convergence. Blue components represent standard NSGA‐II steps, while red marks the newly developed strategies.

Figure 5 depicts the inverse design strategy based on NSGA‐II‐PMCP workflow. NSGA II performs non dominated sorting on the population (i) and selects parent microstructures for crossover, mutation, and prediction (ii). Selection is performed using crowding distance and binary tournament mechanisms to preserve diversity while promoting high‐quality parents. Once a provisional Pareto frontier is established after 20 generations, the partition monitoring (iii) step identifies segments of the front with steep properties gradients and intensifies search in these “subdomains”, while chaotic perturbations (iv) are introduced when population diversity falls under threshold. Chaotic perturbations are applied to diversify the population and help escape local optima. Elite individuals (v) from each generation are systematically preserved to maintain optimal solutions (see Materials and Methods). Elites are archived, periodically reintroduced to seed new generations and optimization check (vi), ensuring retention of the best‐found designs. This loop iterates until convergence, assessed by stagnation in objective improvement or hypervolume (HV) gain, or until a maximum of 50 generations is reached (all converge).

**FIGURE 6:**
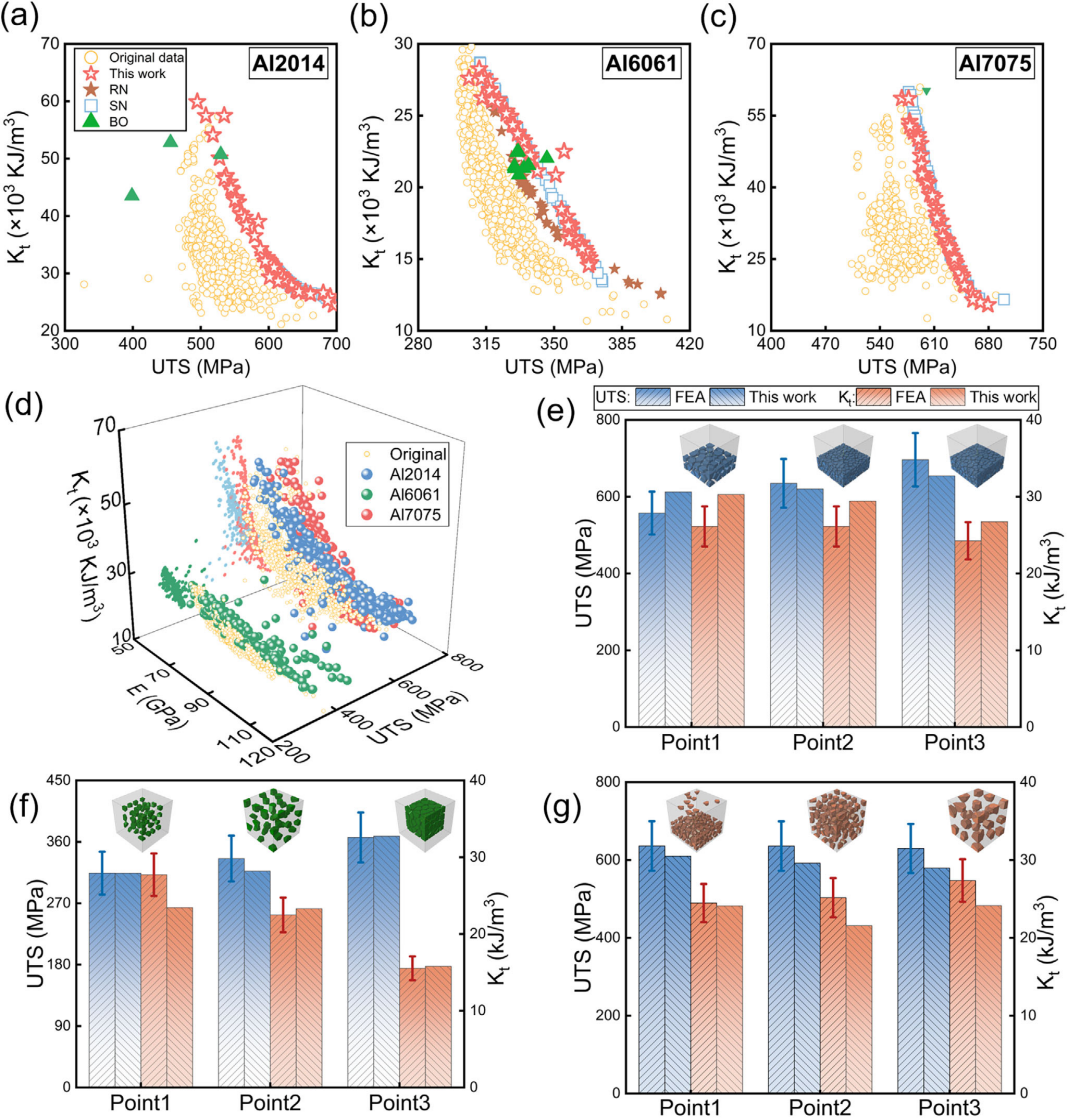
Performances of NSGA‐II‐PMCP optimization. Comparison of the final (50_th_) optimization results of (a) Al2014‐based PRAMCs, (b) Al6061‐based PRAMCs, and (c) Al7075‐based PRAMCs with original dataset, showing the distributions of optimized *UTS* and *K_t_* obtained from Standard NSGA‐II (SN), NSGA‐II‐PMCP (this work), Bayesian optimization (BO), and RFR‐NSGA‐II (RN). (d) Comparison of NSGA‐II‐PMCP optimized results with the original dataset. Comparison of FEA validation and NSGA‐II‐PMCP optimization results for *UTS* and *K_t_* of PRAMCs based on (e) Al2014, (f) Al6061, and (g) Al7075 matrices. Blue and orange histograms represent *UTS* and *K_t_*, respectively, left‐shaded bars indicating FEA results and right‐shaded bars denoting predictions from this work. Error bars represent ±10%. Points 1–3 indicate three representative microstructures randomly selected from this work results for different PRAMCs, with the 3D models above each bar illustrating the corresponding microstructures.

This work benchmarks four algorithms: Standard NSGA‐II (SN), NSGA‐II‐PMCP (this work), Bayesian optimization (BO), and RFR‐NSGA‐II (RN) [70]. Convergence analysis (Figure S10e–f) shows that NSGA‐II‐PMCP increases the HV of Al7075‐based PRAMCs by 68.95% relative to SN, underscoring its superior Pareto‐front quality and diversity. During optimization, NSGA‐II‐PMCP drives the solutions from low‐*UTS* and *K_t_* regions toward higher *UTS* and *K_t_*, whereas the standard NSGA‐II tends to evolve from unrealistically high *UTS*–*K_t_* regions back toward the feasible design space defined by the initial dataset (Figure S11). We then focus on comparing the *UTS* and *K_t_* at the 50_th_ final iteration across four methods for Al2014‐ (Figure 6a), Al6061‐ (Figure 6b), and Al7075‐based PRAMCs (Figure 6c). After convergence, NSGA‐II‐PMCP consistently delivers broader and higher‐quality Pareto fronts (the coverage of the optimized solution set): for Al2014‐based PRAMCs (Figure 6a) its solution set spans a wider and more continuous *UTS*–*K_t_* region than SN, indicating superior coverage even though both methods reach similar upper‐right limits, and markedly surpasses BO; for Al6061‐based PRAMCs (Figure 6b) although RN and BO achieve comparable upper‐right solutions, NSGA‐II‐PMCP exhibits visibly broader distribution and fuller Pareto coverage, clearly outperforming RN and BO; and for Al7075‐based PRAMCs (Figure 6c) its overall Pareto spread significantly exceeds BO, while maintaining comparable high‐performance points to SN. Across all three alloys‐based PRAMCs, the advantage of NSGA‐II‐PMCP achieves the strongest overall trade‐off performance characterized by wider Pareto coverage, higher HV, and more balanced exploration of the feasible high‐performance region. Figure 6d presents a scatter plot comparing the *UTS*, *K_t,_* and corresponding *E* values of the PRAMCs optimized by the NSGA‐II‐PMCP strategy against those from the original dataset (represented as original in the figure), with different colors representing different PRAMCs. It can be observed that the optimized PRAMCs achieve significantly higher *UTS* and *K_t_* values compared to the original data, validating the effectiveness of NSGA‐II‐PMCP. In summary, the runtime of RFR‐NSGA‐II [70] is substantially longer, whereas NSGA‐II‐PMCP completes within ten minutes (Figure S11d). Across all PRAMCs, this work produces broader and higher‐quality Pareto fronts. For Al2014‐based PRAMCs, this work yields a broader and higher‐quality Pareto front than SN and BO. For Al6061‐based PRAMCs, this work's properties range significantly exceeds those of BO and RN. Notably, for Al7075‐based PRAMCs the algorithm increases the HV by 68.95% relative to SN. Overall, NSGA‐II‐PMCP combines computational efficiency with the best *UTS*–*K_t_* trade‐offs, driven by its elite‐retention and targeted‐evaluation mechanisms that concentrate search effort in high‐performance regions.

To validate predictive accuracy, we select three representative microstructures from each PRAMC for FEA validation and compared NSGA‐II‐PMCP predictions with FEA results for *UTS* and *K_t_* (Figure 6e–g). For each alloy, the comparison is shown as bar charts with the corresponding 3D microstructures displayed above each bar: for Al2014 (Figure 6e) three high‐*UTS* optimized microstructures are randomly chosen; for Al6061 (Figure 6f) we select optimized microstructures with low, medium and high *UTS*; and for Al7075 (Figure 6g) we examin three distinct particle‐distribution configurations (laminate, uniform, cluster). Figure 6e–g demonstrates close agreement between FEA‐test and predicted *UTS* and *K_t_*, with all *UTS* errors remaining within 10% and *K_t_* deviations staying close to 10%. This trend matches the surrogate performance of BPNN‐CL, which attains R^2^ = 0.987 for *UTS* and R^2^ = 0.948 for *K_t_*; the slightly lower R^2^ for *K_t_* reflects its greater sensitivity to microstructural heterogeneity and thus larger predictive variance. Overall, the strong agreement across metrics confirms the reliability and robustness of the proposed prediction‐ optimization framework.

This framework demonstrates that matrices, interfaces, and reinforcements synergistically govern the mechanical properties of PRAMCs. Matrix parameters *A* and *n* are the primary drivers of the *UTS*–*K_t_* trade‐off, while reinforcement volume fraction and configuration are the dominant control factors. For high‐strength applications (e.g., surgical blades, satellite brackets, drone components), Al7075 or Al2014 matrices combined with laminated, network, or micro/nano‐hybrid architectures maximize *UTS* and promote crack deflection to enhance toughness. Conversely, when toughness is prioritized (e.g., automotive panels, protective sports gear), lower reinforcement volume fraction and a uniform distribution improve fracture resistance. Beyond co‐optimizing *UTS* and *K_t_*, our BPNN‐CL and NSGA‐II‐PMCP loop delivers both high accuracy and rapid design iterations, offering a practical paradigm for targeted 3D microstructure design.

### 2.6 NSGA‐II‐PMCP for Inverse Design

Inverse design aims to determine microstructural parameters that can produce prescribed mechanical properties. In this work, the inverse design performance of NSGA‐II‐PMCP was examined using three aluminum alloy composite systems. For each system, a target combination of *UTS* and *K_t_* was specified, and the corresponding microstructural parameters predicted by NSGA‐II‐PMCP were further evaluated by finite element analysis. In addition, the Pareto solutions were analyzed to examine the non‐uniqueness of the inverse design problem and its relevance to engineering design.

The target properties and inverse design results for the Al2014‐, Al6061‐, and Al7075‐based composites are summarized in Table 3. For the Al2014‐based composite, the target values were *UTS* = 591.5 MPa and *K_t_* = 33.2 × 10^3^ kJ/m^3^. NSGA‐II‐PMCP predicted a reinforcement volume fraction of 13% and a particle diameter of 12 µm. The FEA verification gave a *UTS* of 530.4 MPa, corresponding to an error of 10.3%, while the error in *K_t_* was only 0.16%. This result indicates that the framework can provide a reasonable inverse mapping between the prescribed strength–toughness target and the microstructural parameters for the Al2014‐based composite. For the Al6061‐based composite, the target values were *UTS* = 333.2 MPa and *K_t_* = 23.4 × 10^3^ kJ/m^3^. The predicted microstructure had a reinforcement volume fraction of 7% and a particle diameter of 11 µm. The FEA results showed errors of 7.4% for *UTS* and 4.6% for *K_t_*, both within 10%. Among the three cases, this system showed the most balanced agreement between the target and verified properties. Although the Al6061 matrix has a relatively lower strength, the framework still identified a feasible design at a low reinforcement content, suggesting that the BPNN‐CL surrogate model can maintain acceptable predictive performance in this design region. For the Al7075‐based composite, the target values were *UTS* = 629.7 MPa and *K_t_* = 27.4 × 10^3^ kJ/m^3^. The optimized microstructure had a reinforcement volume fraction of 16% and a particle diameter of 15 µm. The FEA verification error was 8.7% for *UTS* and approximately 13.3% for *K_t_*. The relatively larger deviation in *K_t_* may be related to the sensitivity of toughness to matrix plastic deformation, crack propagation paths, and particle/matrix interfacial fracture behavior. Compared with *UTS*, *K_t_* is affected by more local microstructural features, which may increase the uncertainty of inverse prediction.

**TABLE 3:** Reverse design properties comparison.

Matrix	Target‐*UTS* (MPa)	Target‐*K_t_* (×10^3^ kJ/m^3^)	Volume fraction (%)	Diameter (µm)	FEA‐*UTS* (MPa)	Error (%)	FEA‐*K_t_* (×10^3^ J/m^3^)	Error (%)
Al2014	591.52	33.23	13	12	530.39	10.33	33.18	0.16
Al6061	333.20	23.44	7	11	310.31	7.38	24.57	−4.59
Al7075	629.68	27.36	16	15	579.27	8.70	24.15	13.29

Overall, the *UTS* errors of the three systems were all within 11%, while the *K_t_* errors were mostly within an acceptable range. These results show that NSGA‐II‐PMCP can generate feasible microstructural designs for different PRAMCs under prescribed strength–toughness targets.

To examine the non‐uniqueness of the inverse design problem, Pareto‐optimal solution sets were further analyzed for the three alloy systems. Table S11 lists nine representative inverse design solutions, with three solutions selected for each alloy. In each alloy group, the predicted *UTS* and *K_t_* values are close to one another, while the corresponding microstructural parameters show clear differences in reinforcement configuration, particle diameter, and volume fraction. For example, the selected Al2014 solutions differ by less than 2% in *UTS* and less than 9% in *K_t_*. The Al6061 and Al7075 solutions also show very small variations in *UTS*, within 0.5% and 1%, respectively. These results indicate that similar mechanical properties can be achieved by different microstructural designs, confirming the non‐unique nature of the inverse mapping between target properties and microstructural parameters.

The form of this non‐uniqueness varies among the three alloy systems. For the Al2014‐based PRAMCs, comparable strength–toughness combinations can be obtained from micro/nano, laminated, and uniform reinforcement configurations, with particle diameters ranging from 8 to 18 µm. This suggests that the Al2014 system has a relatively wide design space for achieving similar target properties. For the Al6061‐based composites, the selected solutions include cluster, micro/nano, and laminated configurations, but their predicted *UTS* values remain very close. This result implies that different reinforcement arrangements may lead to similar global strength responses in this system, although their effects on local damage evolution may still differ. In the Al7075‐based composites, two micro/nano solutions with similar configurations but different particle diameters, together with a network configuration, provide comparable mechanical properties.

From an engineering perspective, the non‐uniqueness of inverse design can provide useful design flexibility. When several microstructures satisfy similar strength–toughness requirements, the final selection does not need to rely only on mechanical performance. Other factors, such as manufacturing feasibility, particle size availability, volume fraction control, and processing compatibility, can also be considered. In this sense, the Pareto‐optimal solution set offers a practical design space rather than a single fixed answer. Compared with a single‐objective optimization strategy, NSGA‐II‐PMCP is able to retain multiple feasible solutions, which is beneficial for subsequent engineering decision‐making.

### 2.7 Experimental Verification

After obtaining the inverse optimization and design results, two uniform PRAMCs, namely SiCp/Al2014 and SiCp/Al6061, were fabricated for experimental validation. For the SiCp/Al2014 composite, SiC particles with a diameter of 12 µm and a volume fraction of 13% were adopted to verify the inverse design value. For the SiCp/Al6061 composite, the SiC particle diameter and volume fraction were set to 8 µm and 5%, respectively, to validate the inverse optimization value. These two samples for different matrix alloys and were used to assess the consistency between the optimization predictions and the experimental results.

**FIGURE 7:**
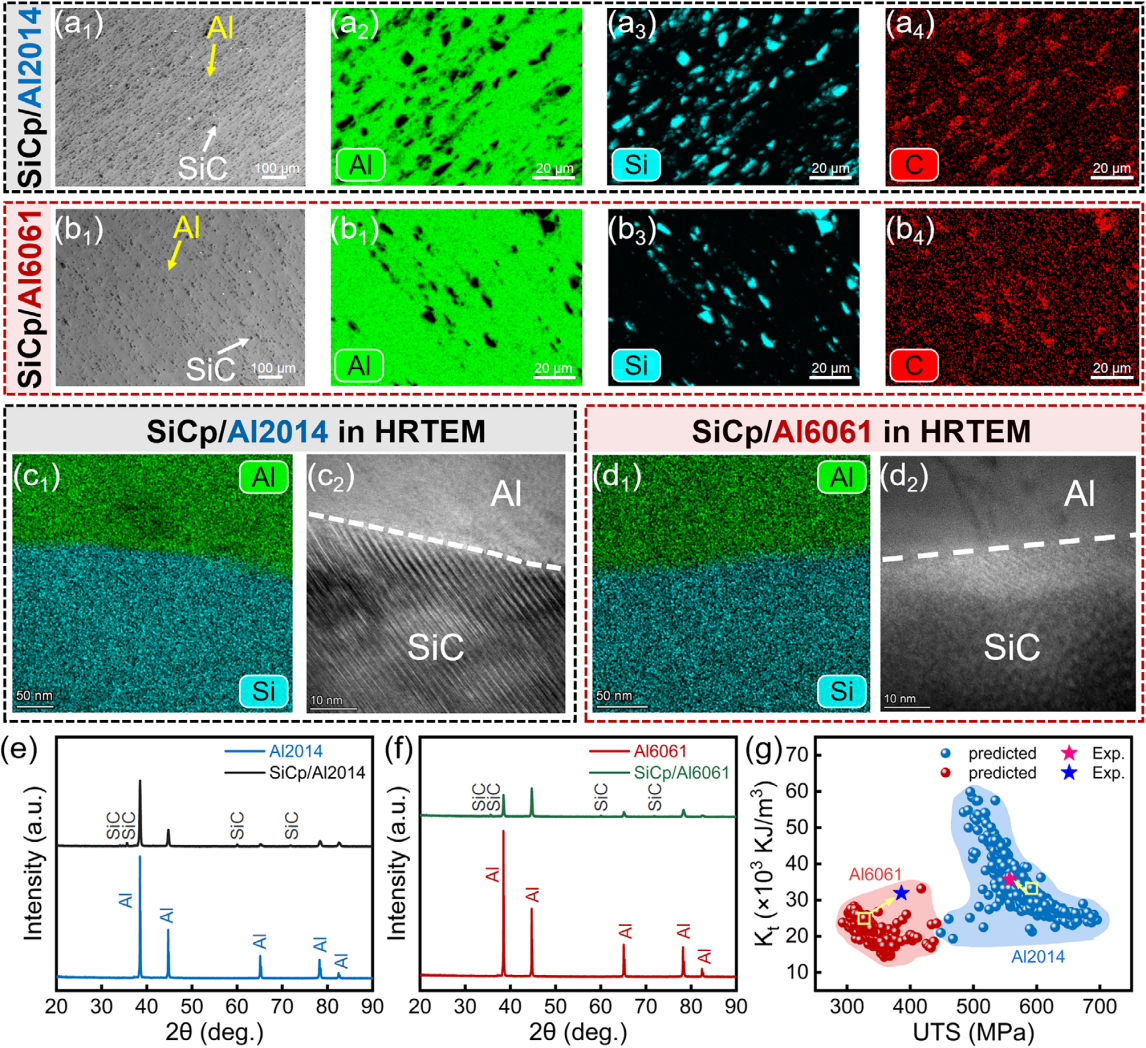
Microstructural characterization and experimental validation of optimized SiCp/Al2014 and SiCp/Al6061 composites. (a_1_–b_4_), BSE and EDS elemental maps of the optimized composites: (a_1_–a_4_), SiCp/Al2014 containing 13 vol.% SiC particles with an average diameter of 12 µm; (b_1_–b_4_), SiCp/Al6061 containing 5 vol.% SiC particles with an average diameter of 8 µm. The corresponding panels show BSE morphology and elemental distributions of Al, Si, and C. (c_1_–d_2_), interfacial characterization of the optimized composites. (c_1_), (c_2_), EDS mapping and HRTEM image of the SiC/Al2014 interface; (d_1_), (d_2_), EDS mapping and HRTEM image of the SiC/Al6061 interface, showing the elemental distribution and local atomic‐scale interfacial structure. (e), (f), XRD patterns of the matrix alloys and corresponding composites: (e), Al2014 and SiCp/Al2014; (f), Al6061 and SiCp/Al6061. (g), comparison among the original dataset, NSGA‐II‐PMCP optimized results and experimental validation results.

The Backscattered Electron (BSE), Energy‐Dispersive x‐ray Spectroscopy (EDS), High‐Resolution Transmission Electron Microscopy (HRTEM), x‐ray Diffraction (XRD), and comparison are shown in Figure 7a_1_–a_4_ for SiCp/Al2014 representative case, while Figure 7b_1_–b_4_ correspond to SiCp/Al6061. The low‐magnification BSE images show no obvious large pores, penetrating cracks, or severe particle agglomeration in either composite. The EDS elemental maps further show the distribution of the particles and matrix. Al is continuously distributed in both composites, indicating that the matrix phase remains connected. Si is mainly concentrated in the particle‐like regions, which correspond to the dark regions in the BSE images and can be identified as SiC reinforcements. Compared with SiCp/Al6061, the Si‐rich regions in SiCp/Al2014 are more densely distributed, consistent with its higher SiC volume fraction. In the SiCp/Al6061 composite, the SiC particles are relatively more dispersed, although local enrichment can still be observed. To examine the local interfacial state, EDS mapping and HRTEM observations were further performed. In the SiCp/Al2014 composite, the EDS map in Figure 7c_1_ shows a clear compositional contrast between the Si‐rich particle region and the surrounding Al2014 matrix. No obvious interfacial discontinuity or large‐scale elemental segregation is observed at the mapped scale. In Figure 7c_2_, the Al2014 matrix and SiC particle can be distinguished on both sides of the interface. The SiC region shows regular lattice fringes, indicating that the SiC particles retain a crystalline structure after processing. The interface appears relatively continuous, without obvious pores, cracks, or debonding in the observed area. Similar features are observed in the SiCp/Al6061 composite. The EDS map in Figure 7d_1_ shows a compositional contrast between the SiC and the Al6061 matrix, and the matrix remains continuous around the particle. These observations indicate that SiC particles can be incorporated into both Al2014 and Al6061 matrices with no obvious interfacial defects at the observed scale.

Figure 7e,f show the XRD patterns of the SiCp/Al2014 and SiCp/Al6061 together with their corresponding matrix alloys. The Al2014 and Al6061 matrices mainly show characteristic diffraction peaks of the Al phase. After the addition of SiC, SiC diffraction peaks appear in both composites, indicating the presence of SiC in the Al matrices. No obvious additional diffraction peaks are detected, suggesting that no large amount of coarse reaction products or detectable secondary phases formed under the present processing conditions. Figure 7g shows the comparison of NSGA‐II‐PMCP experiment results, optimized results, and original dataset. For the SiCp/Al6061 composite, the optimized *UTS* was 328 MPa, while the experimental value reached 386 MPa, which is approximately 17.7% higher than the optimized value. The experimental toughness was also 27.9% higher than the optimized value. This suggests that the prediction for the SiCp/Al6061 system is relatively conservative. The relatively dispersed SiC distribution and the continuous SiCp/Al interface observed in the microstructural characterization may contribute to the improved experimental performance. For the SiCp/Al2014 composite, the experimental *UTS* was 557 MPa, which is 5.8% lower than the optimized result. This indicates that the experimental strength did not fully reach the predicted value. The deviation may be associated with the higher sensitivity of the high‐strength Al2014 matrix to particle distribution, local defects, and interfacial stress concentration. During processing, local SiC enrichment, flow‐induced heterogeneity, and nonuniform micro‐deformation may reduce the measured strength compared with the ideal optimized prediction. However, the experimental toughness of SiCp/Al2014 was 7.4% higher than the optimized result, indicating improved energy absorption during tensile deformation.

Overall, the experimental results show reasonable consistency with the optimized predictions, although the two matrix systems exhibit different deviation characteristics. For SiCp/Al6061, both strength and toughness exceed the optimized values, suggesting a conservative prediction for this system. For SiCp/Al2014, the strength is slightly lower than the prediction, while the toughness is higher. These results indicate that the optimization framework can provide useful guidance for experimental design. At the same time, further experimental data from different material systems and processing conditions are still needed to improve the predictive accuracy and general applicability of the model.

## 3 Conclusions

In this study, we establish a state‐of‐the‐art framework integrating BPNN‐CL and NSGA‐II‐PMCP for the inverse design of different HSMMCs. For prediction, R^2^ values for *UTS* and *K_t_* exceed 0.98 and 0.94, respectively. Against seven mainstream static methods, BPNN‐CL is consistently the best. External validation on experiments shows most *UTS* and *E* predictions fall within ±10% of reported values, even for some absent from training, which demonstrates meaningful extrapolative power. For inverse exploration, the optimized *UTS*‐*K_t_* Pareto frontiers for Al2014, Al6061, and Al7075‐based PRAMCs all perform better than Bayesian optimization. Specifically, Al7075‐based PRAMCs show 68.95% higher HV than standard NSGA‐II. Also, Al6061‐based PRAMCs designs achieve better *UTS*‐*K_t_* with faster convergence than RFR‐NSGA‐II. Furthermore, the Al6061‐based PRAMCs achieve superior *UTS* and *K_t_* with accelerated computational efficiency compared RFR‐NSGA‐II. This efficiency gain stems from the direct, parallel optimization of *UTS* and *K_t_* using the NSGA‐II‐PMCP optimizer, which eliminates the need for repeated comparisons with earlier datasets, thereby substantially reducing computation time. Overall, the NSGA‐II‐PMCP framework not only enhances Pareto front coverage but also significantly reduces the optimization time. Future work will explicitly incorporate Hall–Petch‐related descriptors, such as the matrix grain size, to quantify the contribution of grain‐size strengthening to microstructural heterogeneity. The effect of boundary conditions will also be investigated by comparing free‐surface and periodic boundary conditions for representative microstructural models, which will help define the applicability of the present modeling framework more clearly. Moreover, since the simulation database contains the complete stress–strain curve for each representative microstructural model, future work will extend the present feature‐level prediction to curve‐level constitutive modeling, enabling direct prediction of full stress–strain responses. In addition, strongly anisotropic reinforcement architectures may require additional orientation‐ and topology‐related descriptors in future studies. Looking forward, the approach can be extended to diverse domains such as solid mechanics, marine engineering, and microrobotics, enabling the inverse design of customized, high‐performance composites.

## 4 Materials and Methods

### 4.1 Datasets Generation

The microstructural datasets were generated using Python and Neper and then imported into Abaqus 2022. Displacement‐controlled uniaxial tensile loading was applied, with one end constrained to suppress rigid‐body motion and the opposite end subjected to a prescribed tensile displacement. For the heat‐treatable aluminum matrices considered in this work, the matrix strength is affected by alloy composition, heat‐treatment condition, solid‐solution strengthening, precipitation strengthening, and grain‐size strengthening. In the present representative microstructural modeling framework, these strengthening mechanisms are not explicitly modeled as separate micro‐ or nanoscale phases. Instead, the aluminum matrix is treated as an equivalent continuum, and its plastic flow behavior is described using the Johnson–Cook constitutive model. The corresponding descriptors D3–D6 are obtained from experimentally calibrated constitutive responses of each alloy system under its actual processing and heat‐treatment condition. Therefore, the effects of solute atoms, precipitates, and matrix grain structure are not ignored, but are implicitly reflected in the calibrated matrix flow‐stress curve and the resulting constitutive parameters.

Specifically, interface productions were systematically included (the reaction principles in Principle S1). The Johnson–Cook law was used on the matrix, and Hillerborg's fracture energy concept (*G_f_*) was introduced [91] (Principle S2) to address mesh sensitivity. Mesh convergence and toughness integral were introduced in Principle S3. Material properties were literature‐sourced (Table S1), and benchmark details were listed in Table S2. Microstructure generation/meshing used an Intel Xeon Platinum (32 cores, 128 GB RAM). Abaqus simulations ran on AMD EPYC (256 GB RAM).

### 4.2 Key Descriptors Screening

We applied the Mantel test (vegan R package) to quantify the correlations between descriptors and properties [92]. Euclidean distance matrices were constructed. Partial Mantel correlations were then calculated to evaluate the strength and statistical significance of these associations, while adjusting for potential confounding factors (Principle S4). RFR and SHAP (Principles S5 and S6) analyses were also employed to mitigate multicollinearity within the high‐dimensional feature space [93, 94] with data split into training, testing, and validation sets in a 5:2:3 ratio. Because geometric heterogeneity may introduce anisotropy, we evaluated the directional responses of representative microstructural models under X‐, Y‐, and Z‐direction tensile loading. The stress–strain curves were generally close in the elastic and peak‐strength stages, especially for uniform and clustered configurations, indicating a statistically near‐isotropic response for *E* and *UTS* within the present design space. Directional differences mainly appeared in the post‐peak fracture stage, particularly for networked and laminated configurations, suggesting that reinforcement topology primarily affects damage evolution and crack‐path selection. Therefore, particle configuration, diameter, volume fraction, and diameter variance were retained as the main geometric descriptors, while explicit orientation descriptors will be considered in future work for strongly anisotropic systems. Anisotropy analysis is elaborated in detail in Principle S3. All feature selection were used an Intel Xeon Platinum (32 cores, 128 GB RAM).

### 4.3 BPNN‐CL for Forward Prediction

BPNN‐CL was implemented in TensorFlow 2.x. Training employs the Adam optimizer, MSE loss, a batch size of 32, and callbacks to prevent overfitting. BPNN weights were transferred into two incremental stages, cutting trainable parameters. The parameter update rule followed Equation (1). Here, θ_*t*_ denotes the network parameters (including weights and biases) at the *t_th_* iteration, and η is the learning rate. ${\hat{m}}_{t}$ and $\hat{v_{t}}$ represent the bias‐corrected first and second moment estimates of the gradients, respectively, while *ε* is a small constant introduced to prevent division by zero. The raw first‐order moment *m_t_* and second‐order moment *v_t_* are computed using exponential moving averages of the gradients *g_t_* = ∇_θ_L_*t*_, following the update rules: *m_t_* = β_1_ *m*_*t* − 1_ + (1 − β_1_)*g_t_* and $v_{t}=\beta_{2}v_{t-1}+\left(1-\beta_{2}\right)g_{t}^{2}$. ε is a small positive constant introduced to stabilize the denominator in the Adam update and prevent numerical issues such as division by zero. For continual learning, parameter optimization meets Equation (2). Where θ_*BPNN*_ is a BPNN initialization, and *T_i−1_* is the iteration at which training of stage *i−1* stops.

(1) $$\theta_{t+1}=\theta_{t}-\eta \frac{\frac{\beta_{1}m_{t-1}+\left(1-\beta_{1}\right)g_{t}}{\left(1-\beta_{1}^{t}\right)}}{\left(\sqrt{\frac{\beta_{2}v_{t-1}+\left(1-\beta_{2}\right)\left(g_{t}⊙g_{t}\right)}{1-\beta_{2}^{t}}}+\varepsilon \right.},g_{t}=\nabla_{\theta }L_{t}\left(\theta_{t}\right)$$

(2) $$\theta_{0}^{\left(1\right)}=\theta_{BPNN},\theta_{0}^{\left(i\right)}=\theta_{T_{i-1}}^{\left(i-1\right)},i=2,3$$

Detailed principles are listed in Principles S7 and S8. Hyperparameters of the BPNN‐CL can be found in Table S5, with the model architectures in Table S6. All training, testing, and validation were used an Intel Xeon Platinum 8383 CPU@2.7GHZ.

### 4.4 NSGA‐II‐PMCP for Inverse Optimization

Implemented using pymoo, the NSGA‐II‐PMCP integrated the BPNN‐CL as a surrogate model. Partition monitoring discretized the targeted *UTS*‐*K_t_* into 5 × 5 grids space. If no new grid coverage occurs for 20 consecutive generations, chaotic perturbation was activated, perturbing the bottom 5% of the population using a logistic map to escape local optima. Concurrently, an Elite Memory Module archives non‐dominated solutions over a 100‐generation rolling window. During stagnation, elites were reinjected or replaced weak individuals to enhance search efficiency. Algorithm parameters included a population size of 50, simulated binary crossover (SBX) with a probability of 0.9 and distribution index η = 15, plus polynomial mutation at adaptive rate with η = 20. Initialization leverages 20% archived Pareto‐optimal solutions for accelerated convergence. Diversity preservation in NSGA‐II relied on crowding distance calculation (Equation (3)). Here, *CD(X)* denotes the crowding distance of solution *X*; $X_{next}^{\left(i\right)}$ and $X_{prev}^{\left(i\right)}$ are the neighboring solutions in objective space; *m* is the number of objective functions; boundary solutions in each objective are assigned an infinite crowding distance and $f_{i}^{max}$ and $f_{i}^{min}$ are the maximum and minimum values of the *i_th_* objective within the considered solution set. (details in Tables S9 and S10 and Principle S9).

(3) $$CD\left(X\right)=\sum_{i=1}^{m}\frac{f_{i}\left(X_{next}^{\left(i\right)}\right)-f_{i}\left(X_{prev}^{\left(i\right)}\right)}{f_{i}^{max}-f_{i}^{min}}$$

Convergence was assessed using HV and fitness metrics. The NSGA‐II‐PMCP framework were performed on a computer with Intel Xeon Platinum 8383 CPU@2.7 Gigahertz.

### 4.5 Preparation and Characterization

SiCp/Al2014 and SiCp/Al6061 composites were fabricated by powder metallurgy, including powder blending, compaction, thermal holding, hot extrusion, solution treatment, and artificial aging. SiC particles were mechanically blended with Al2014 and Al6061 alloy powders using a modified powder mixer to obtain composite powders with relatively homogeneous particle dispersion. The blended powders were loaded into a die and compacted at room temperature using a hydraulic press to form green billets. After thermal holding, the billets were hot‐extruded to obtain dense composite specimens. The extruded SiCp/Al2014 composite was solution‐treated at 783 K for 2 h and aged at 438 K for 18 h, while the SiCp/Al6061 composite was solution‐treated at 823 K for 2 h and aged at 448 K for 8 h. The heat‐treated composite samples were used for mechanical testing and microstructural characterization.

The microstructure and phase constitution of the SiCp/Al2014 and SiCp/Al6061 composites were characterized using SEM, EDS, HRTEM, and XRD. The samples were mechanically polished before SEM observation. SEM imaging and EDS analysis were carried out on a ZEISS Sigma 300 field‐emission scanning electron microscope. Secondary‐electron imaging, backscattered‐electron imaging, and energy‐dispersive spectroscopy were used to examine the SiC particle distribution, interfacial morphology, and local elemental composition. HRTEM observations were performed using a Talos F200X transmission electron microscope operated at 200 kV. Phase analysis was conducted using a Rigaku SmartLab 9 kW multifunctional rotating‐anode x‐ray diffractometer.

## Author Contributions

**Zhiyan Zhong**: Writing – original draft, Validation, Methodology, Investigation, Formal analysis, Data curation. **Xueru Zheng**: Validation, Methodology, Investigation. **Xiao Zhou**: Conceptualization, Writing – review & editing, Supervision, Resources, Funding acquisition. **Zhengheng Tao**: Investigation, Methodology. **Wan Han**: Investigation, Methodology. **Jingyi Pan**: Methodology, Investigation. **Dian Wu**: Methodology, Investigation. **Ning Gao**: Methodology, Investigation. **Lei Liu**: Methodology, Investigation. **Zhongyang Wang**: Methodology, Investigation. **Fanchao Meng**: Methodology, Investigation. **Tongxiang Fan**: Writing – review & editing, Supervision, Resources.

## Conflicts of Interest

The authors declare no conflicts of interest.

## Supporting information




**Supporting File**: advs76524‐sup‐0001‐SuppMat.docx.

## Data Availability

The data that support the findings of this study are available from the corresponding author upon reasonable request.
