# Supplementary material for: A Closed‐Loop Framework for Inverse Design: Dynamic Training and Intelligent Optimization for Heterostructured Materials
Source: Adv Sci (Weinh). 2026 Jul 20:e76524. Online ahead of print. doi: 10.1002/advs.76524 (PMC13383155; doi:10.1002/advs.76524)
Supplement: Supplementary file 1 — Supporting File: advs76524‐sup‐0001‐SuppMat.docx. [file ADVS-9999-e76524-s001.docx]

**Supplementary Information for**

**A closed-loop framework for inverse design: dynamic training and intelligent optimization for heterostructured materials**

Zhiyan Zhong ^a, b, #^, Xueru Zheng ^a, #^, Xiao Zhou ^a, c, *^, Zhengheng Tao ^a^, Wan Han ^a^, Jingyi Pan ^a^, Dian Wu ^d^, Ning Gao ^a^, Lei Liu ^a^, Zhongyang Wang ^a, *^, Fanchao Meng ^e^, Tongxiang Fan ^a, *^

*^a^ State Key Lab of Metal Matrix Composites, School of Material Science and Engineering, Shanghai Jiao Tong University, 800 Dong Chuan Road, Shanghai 200240, PR China.*

*^b^ Shanghai Innovation Institute, 699 Hua Fa Road, Shanghai 200240 PR China.*

*^c^ Institute of Medical Robotics, Shanghai Jiao Tong University, Shanghai 200240, PR China.*

*^d^ School of Mathematical Sciences, Peking University, Beijing 100871, PR China.*

*^e^ Institute of Advanced Studies in Precision Materials, Yantai University, 30 Qing Quan Road, Yantai Shandong 264005, PR China.*

^*^Corresponding author: zhouxiao113@sjtu.edu.cn (X. Zhou), zy_wang@sjtu.edu.cn (Z.Y. Wang), txfan@sjtu.edu.cn (T.X. Fan)

^#^ These authors contributed equally to this work.

[Main Parameters symbol explanations in the article](#M1)

List of Figures

**[Figures. S1 | Configurations in datasets](#F1)**

**[Figures. S2 | HSMMCs microstructures and dataset](#F2)**

**[Figures. S3 | Al2014-based particle reinforced AMCs FEM fracture analysis](#F3)**

**[Figures. S4 | Al2024-based particle reinforced AMCs FEM fracture analysis](#F4)**

**[Figures. S5 | Al6061-based particle reinforced AMCs FEM fracture analysis](#F5)**

**[Figures. S6 | Al7075-based particle reinforced AMCs FEM fracture analysis](#F6)**

**[Figures. S7 | The R](#F7)^[2](#F7)^ [and MAPE of random forest regression analysis](#F7)**

**[Figures. S8 | The Mantel test for](#F8) *[E](#F8)*[,](#F8) *[UTS](#F8)* [and](#F8) *[K](#F8)_[t](#F8)_***

**[Figures. S9 | The analysis for BPNN-CL](#F9)**

**[Figures. S10 | The converage of NSGA-II and NSGA-II-PMCP](#F10)**

**[Figures. S11 | The comparison of optimization outcomes](#F11)**

List of tables

**[Tables. S1 | Simulation parameters- interface and reinforcement](#T1)**

**[Tables. S2 |](#T2)** **[Benchmark of the data](#T2)**

**[Tables. S3 | SHAP Feature importance](#T3)**

**[Tables. S4 | Static model regression parameters](#T4)**

**[Tables. S5 | BPNN-CL](#T5)** **[hypeparameters](#T5)**

**[Tables. S6 | BPNN-CL architecture](#T6)**

[**Tables. S7 | The Computational time and memory complexity**](#T7)

**[Tables. S8 |](#T8)** **[Comparison between BPNN-CL and references](#T8)**

**[Tables. S9 | NSGA-II hypeparameters](#T9)**

**[Tables. S10 | NSGA-II-PMCP hypeparameters](#T10)**

**[Tables. S11](#T11)** [|](#T11) **[The non-uniqueness solution](#T11)**

List of principles

**[Principles. S1 | The reaction priciples of Al](#P1)_[4](#P1)_[C](#P1)_[3](#P1)_[, Mg](#P1)_[2](#P1)_[Si and Al](#P1)_[4](#P1)_[Si](#P1)_[3](#P1)_**

**[Principles. S2 | Johnson-Cook model](#P2)**

**[Principles. S3 | FEA principles, Mesh convergence, Toughness integral, influence of specimen-level necking and fracture, and anisotropy analysis](#P3)**

**[Principles. S4 | Mantel Test](#P4)**

**[Principles. S5 | Random forest regression](#P5)**

**[Principles. S6 | SHAP Analysis](#P6)**

**[Principles. S7 | Back Propagation Neural Network](#P7)**

**[Principles. S8 | Continual Learning](#P8)**

**[Principles. S9 | NSGA-II-PMCP](#P9)**

[**Code and model acquisition**](#code)

Main Parameters symbol explanations in the article

| **Parameters** | **Abbreviations** |
| --- | --- |
| Ultimate tensile strength | *UTS* |
| Toughness | *K_t_* |
| Elastic modulus | *E* |
| Heterostructured metal matrix composites | HSMMCs |
| Particle-reinforced aluminum matrix composites | PRAMCs |
| Designed reinforcement architecture without interface products | K1 |
| Reinforcement random architecture without interface products | K2 |
| Reinforcement random architecture with interface products | K3 |
| Fracture strain | D1 |
| Matrix elastic modulus | D2 |
| Yield strength (*A*) | D3 |
| Hardening modulus(*B*) | D4 |
| Hardening exponent(*n*) | D5 |
| Rate hardening parameter(*C*) | D6 |
| Interface volume | D7 |
| Thickness of interface | D8 |
| Al_4_C_3_ ratio* | D9 |
| Mg_2_Si ratio* | D10 |
| Al_4_Si_3_ ratio* | D11 |
| Reinforcements configurations | D12 |
| Reinforcement diameter | D13 |
| Reinforcement volume fraction | D14 |
| Reinforcement diameter variance | D15 |
| Ultimate tensile strength (*UTS*) | P1 |
| Toughness (*K_t_*) | P2 |
| Elastic modulus (*E*) | P3 |
| 1-fold cross-validation | F1 |
| 2-fold cross-validation | F2 |
| 3-fold cross-validation | F3 |
| 4-fold cross-validation | F4 |
| 5-fold cross-validation | F5 |
| The average of five-folds cross-validation | AVG |
| Standard NSGA-II | SN |
| NSGA-II-PMCP | NP (this work) |
| Bayasian optimization | BO |
| Previous work | PW |
| Coefficient of Determination | R^2^ |
| Root Mean Square Error | RMSE |
| Mean Absolute Error | MAE |
| Mean squared error | MSE |
| Mean Absolute Percentage Error | MAPE |

* The ratio refers to the proportion of an individual interfacial product relative to the total interfacial products.

Figures. S1 Configurations in datasets

| **Configurations** | **RVE** |
| --- | --- |
| Network |  |
| Laminated |  |
| Random |  |
| Sphere |  |
| Uniform |  |
| Hybrid |  |
| Cluster |  |

Figures. S2 HSMMCs microstructures and dataset


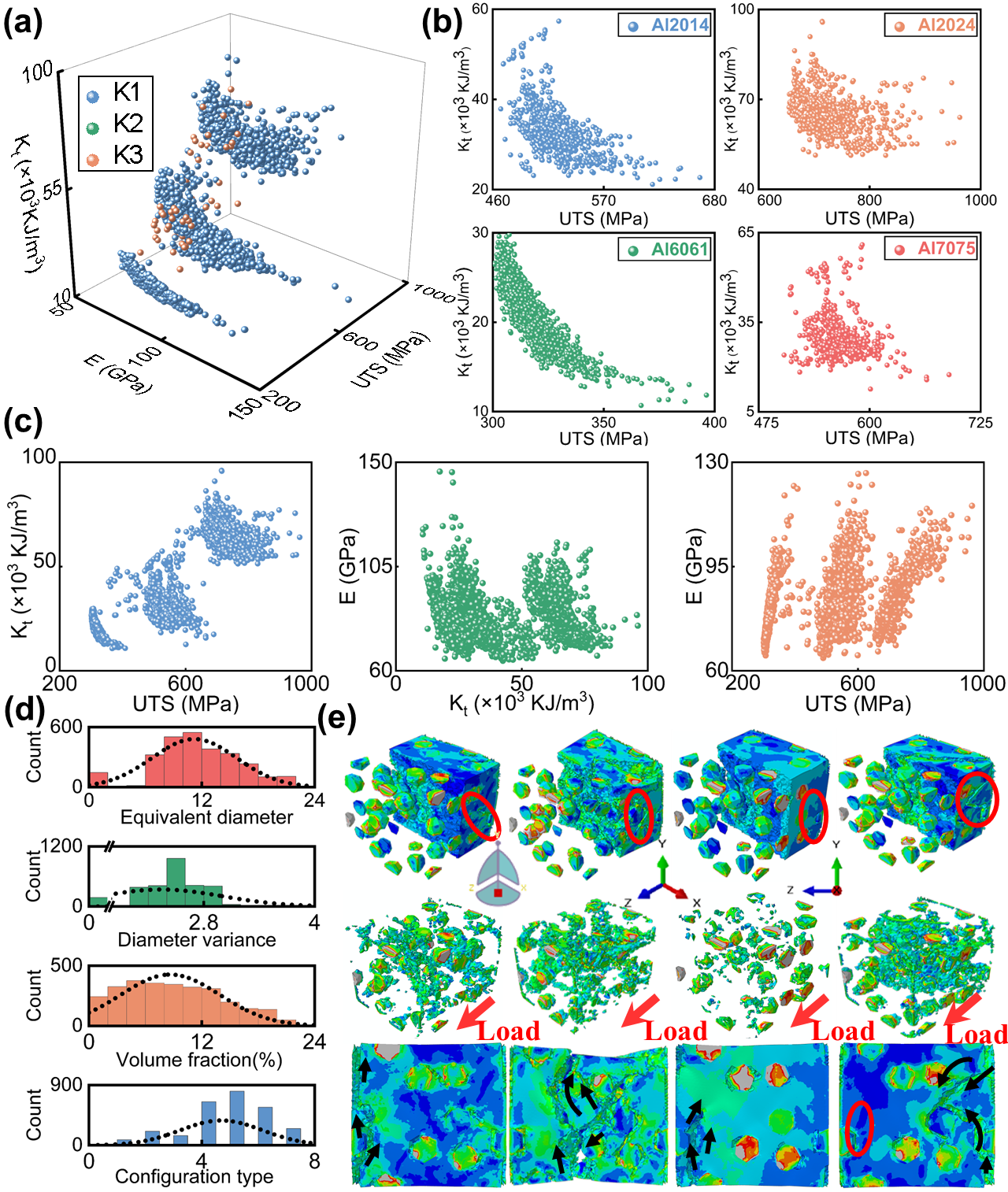


Figures. S2 HSMMCs microstructures and dataset. (a) Scatter plot of *UTS*-*K_t_*-*E* for the dataset. (b) *UTS*-*K_t_* scatter plots for different HSMMCs. (c) Correlation plots of *UTS*-*K_t_-E*. (d)Distribution of different material and structure descriptors. (e) Stress contour plots for random configurations without interface products on Al2014, Al2024, Al6061, and Al7075-based HSMMCs (1st row), highlighting structures subjected to Mises stress exceeding 500 MPa (2nd row) and crack propagation in the ZY view (3rd row).

Figures. S3 Al2014 multi-configuration fracture analysis


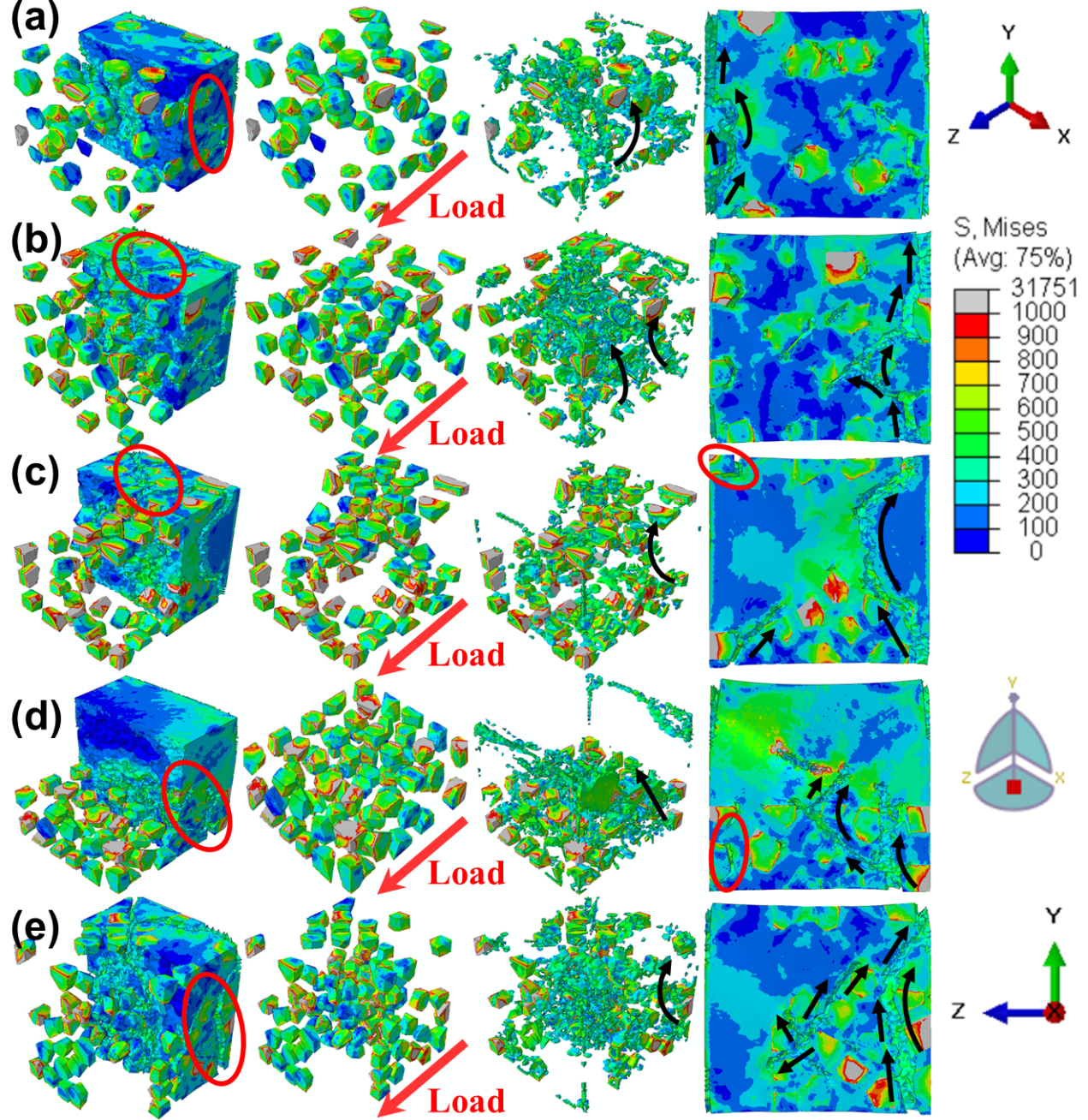
**Figures. S3** **Al2014 multi-configuration fracture analysis**: (a) Random distribution configuration, (b) Uniform distribution configuration, (c) Network distribution configuration, (d) Laminated distribution configuration, (e) Cluster distribution configuration. The subfigures illustrate the overall structure, reinforcement, regions experiencing a bearing stress greater than 500 MPa, and YZ plane fracture analysis.

Figures. S4 Al2024 multi-configuration fracture analysis


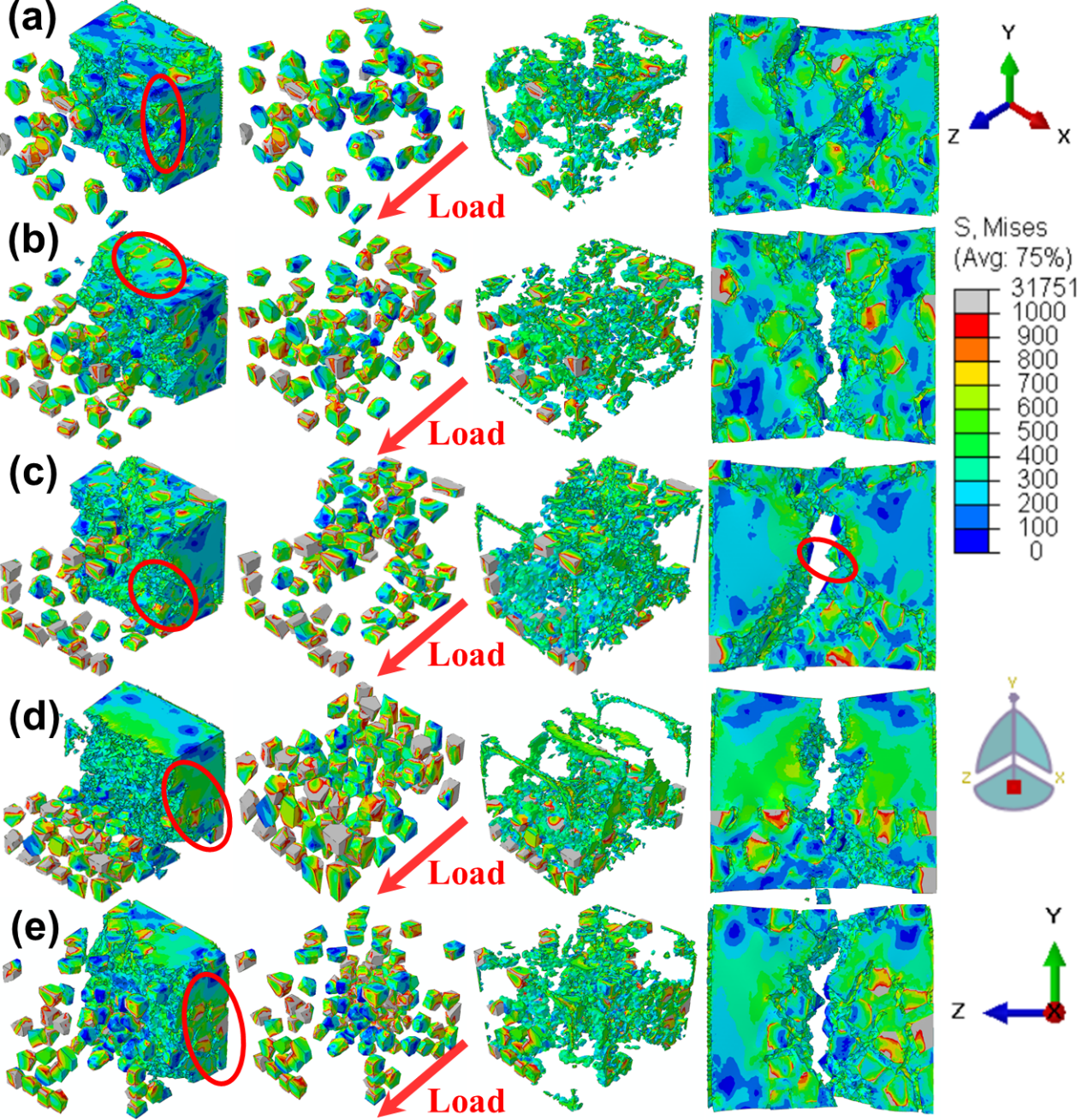


**Figures. S4 Al2024 multi-configuration fracture analysis**: (a) Random distribution configuration, (b) Uniform distribution configuration, (c) Network distribution configuration, (d) Laminated distribution configuration, (e) Cluster distribution configuration. The subfigures illustrate the overall structure, reinforcement, regions experiencing a bearing stress greater than 500 MPa, and YZ plane fracture analysis.

Figures. S5 Al6061 multi-configuration fracture analysis


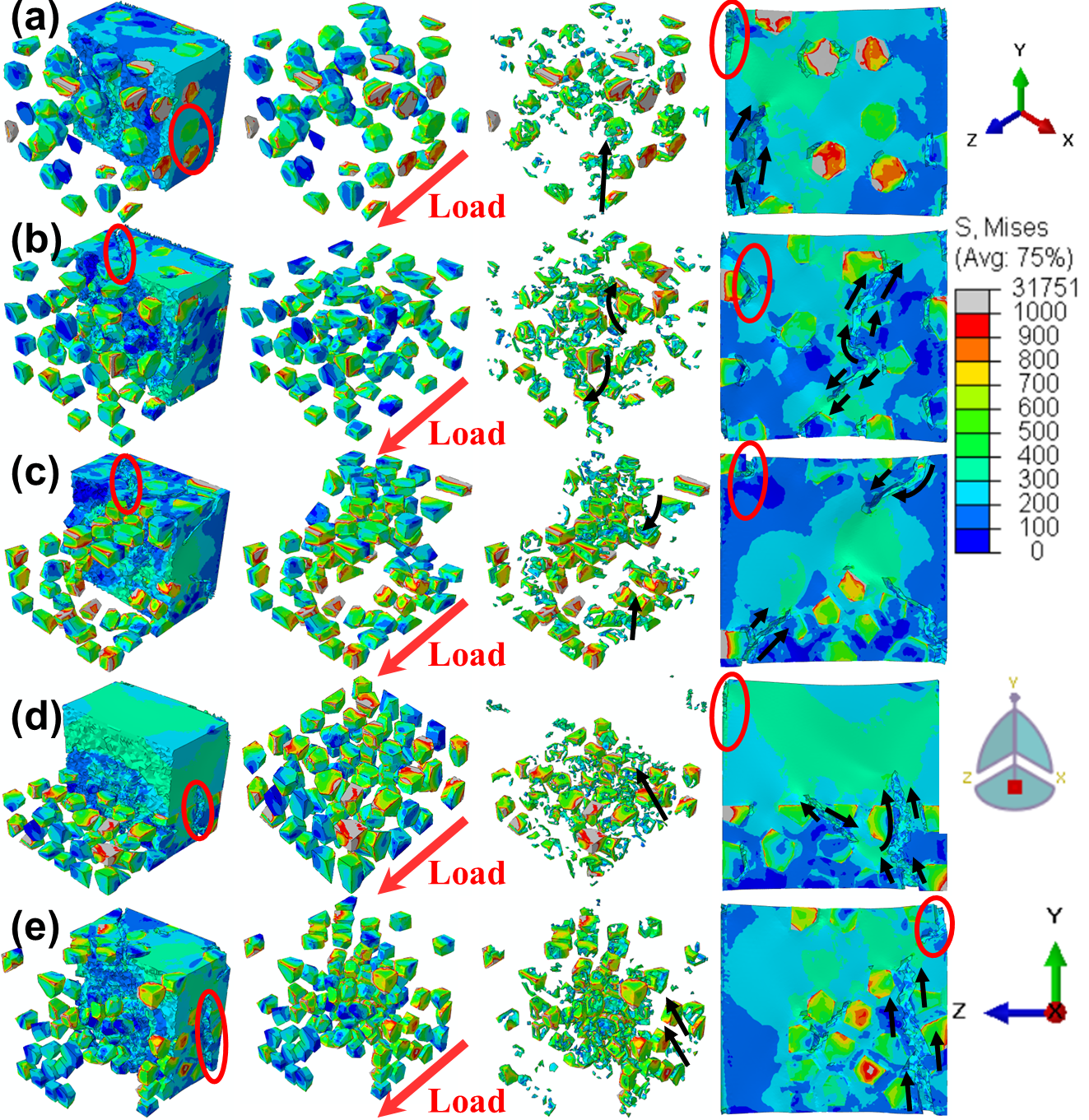


**Figures. S5 Al6061 multi-configuration fracture analysis**: (a) Random distribution configuration, (b) Uniform distribution configuration, (c) Network distribution configuration, (d) Laminated distribution configuration, (e) Cluster distribution configuration. The subfigures illustrate the overall structure, reinforcement, regions experiencing a bearing stress greater than 500 MPa, and YZ plane fracture analysis.

Figures. S6 Al7075 multi-configuration fracture analysis
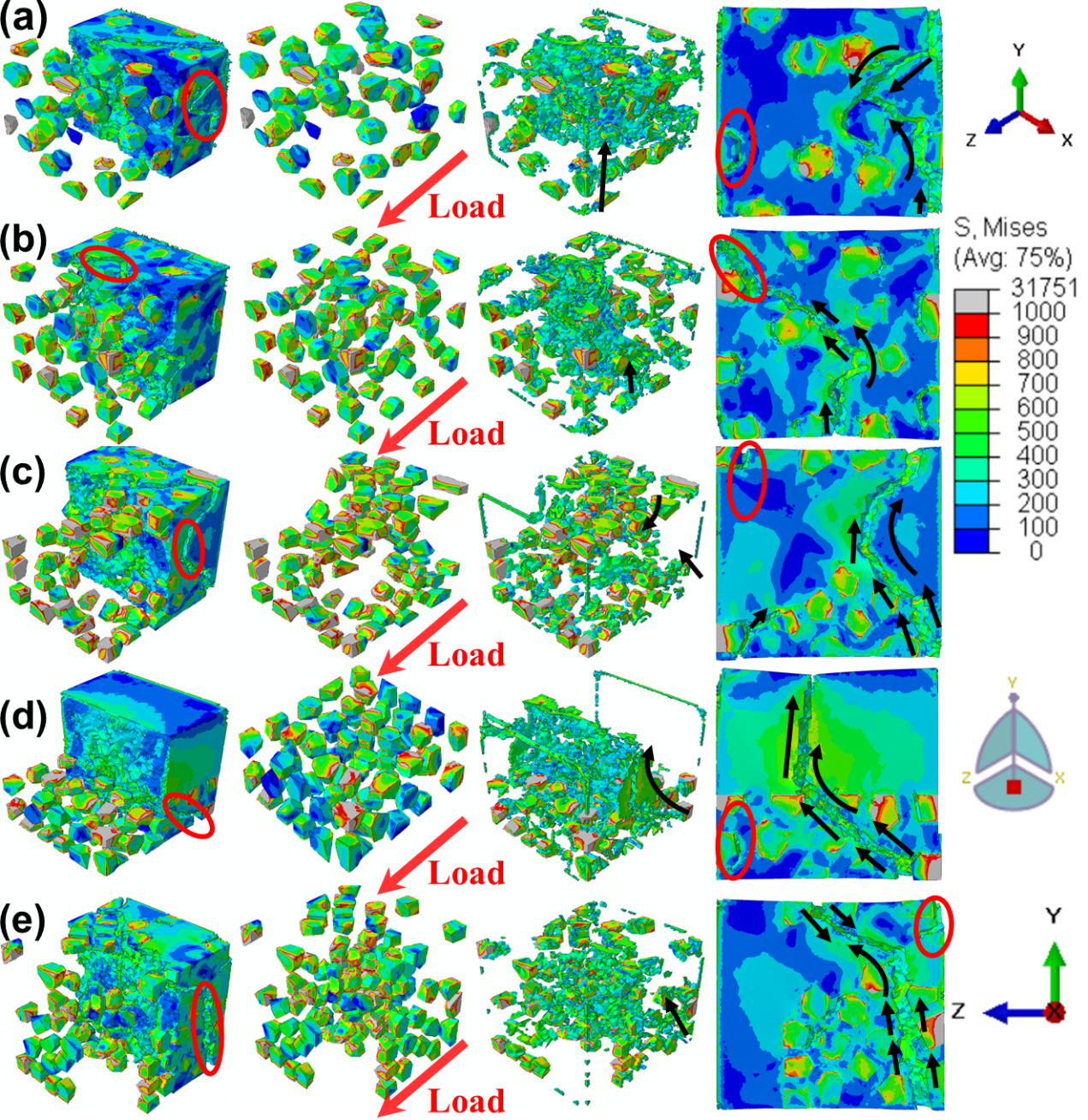


**Figures. S6 Al7075 multi-configuration fracture analysis**: (a) Random distribution configuration, (b) Uniform distribution configuration, (c) Network distribution configuration, (d) Laminated distribution configuration, (e) Cluster distribution configuration. The subfigures illustrate the overall structure, reinforcement, regions experiencing a bearing stress greater than 500 MPa, and YZ plane fracture analysis.

Figures. S7 Random forest regression analysis
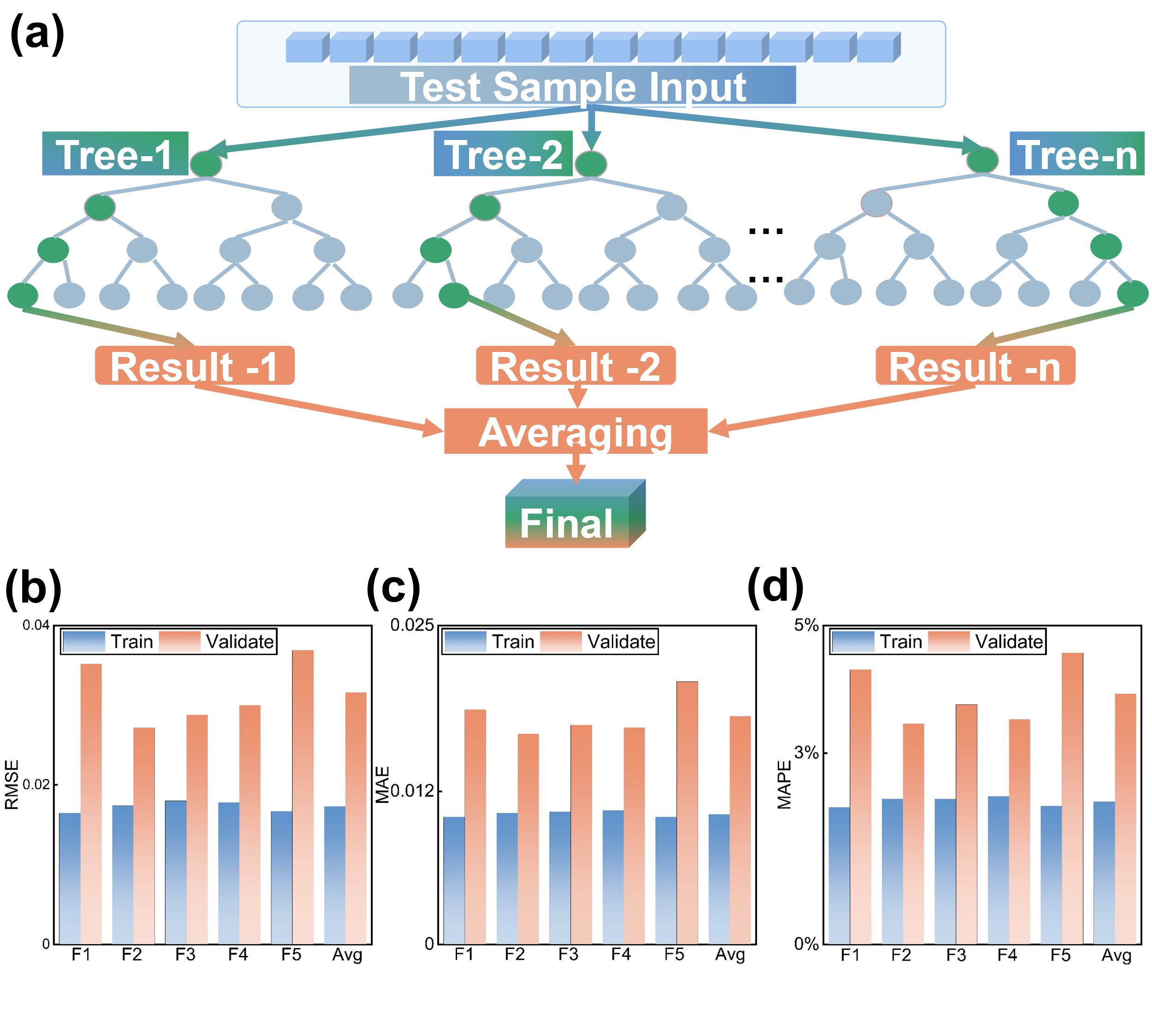


**Figures. S7. Random forest regression analysis**. (a) Schematic illustration of random forest regression principles. (b) The RMSE of test and validation dataset results from five-fold cross-validation. (c) The MAE of test and validation dataset results from five-fold cross-validation. (D) The MAPE of test and validation dataset results from five-fold cross-validation.

Figures. S8 *E*, *UTS* and *K_t_* mantel test and SHAP analysis
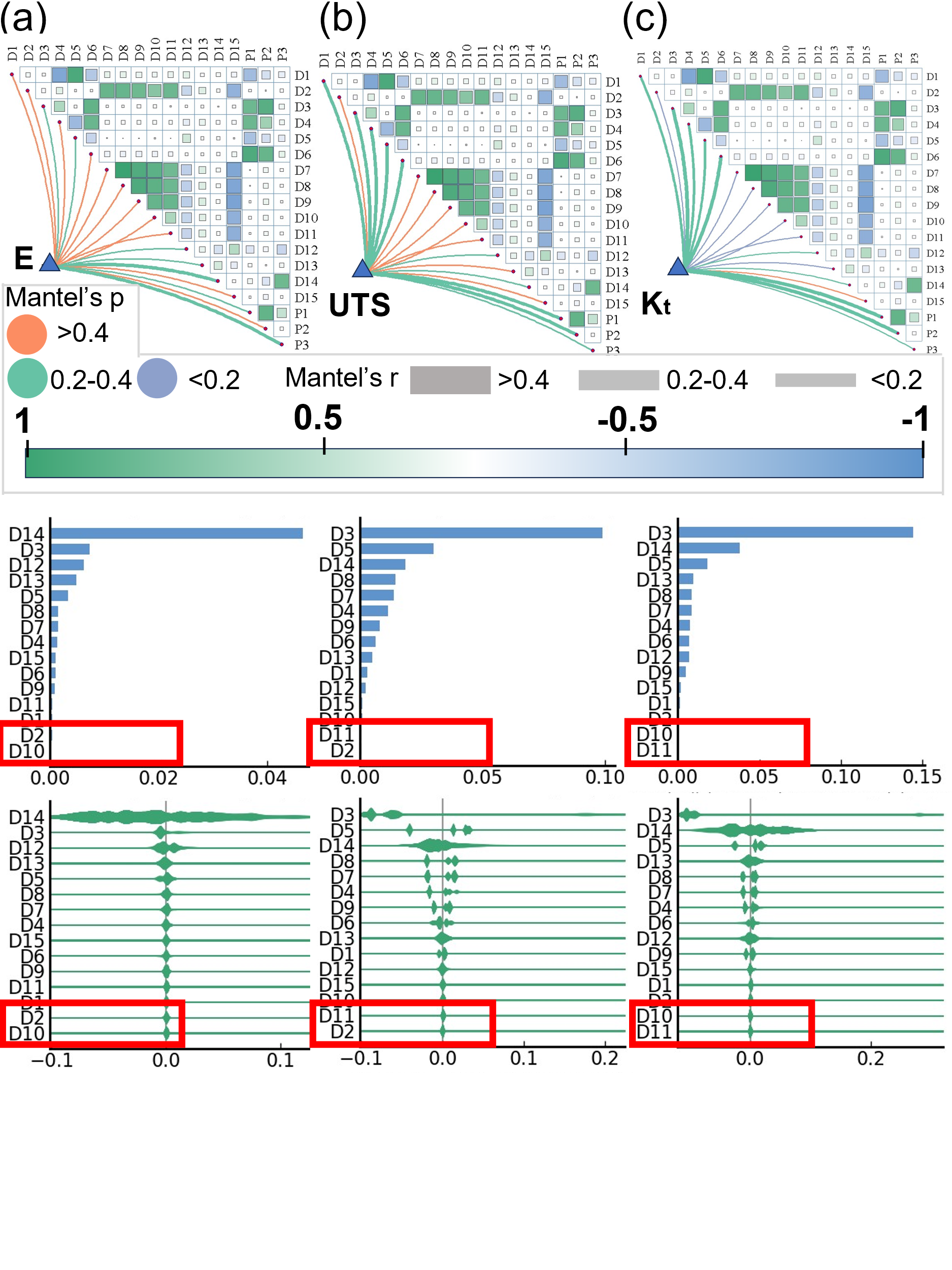


**Figures. S8 *E*, *UTS* and *K_t_* mantel test and SHAP analysis**. (a) Analysis of the influence of matrix-interface-reinforcement parameters on the *E*. (b) Analysis of the influence of matrix-interface-reinforcement parameters on the *UTS.* (c) Analysis of the influence of matrix-interface-reinforcement parameters on the *K_t._*

Figures. S9 BPNN and Continual learning analysis
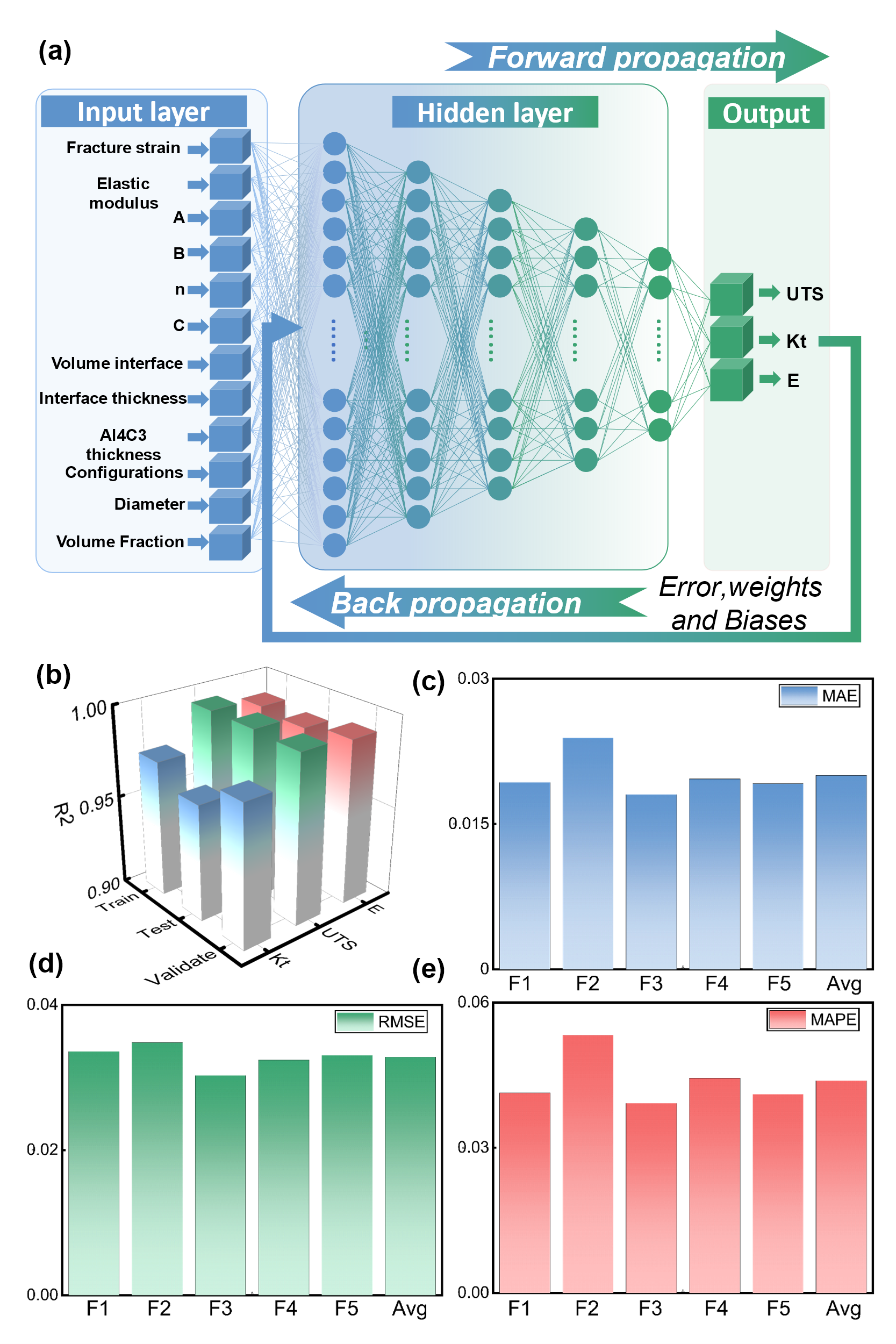


**Figures. S9. BPNN and Continual learning analysis**. (a) Schematic illustration of BPNN principles. (b) *R^2^* values of *UTS*, *K_t_*, and *E* in the training, test, and validation datasets (5:2:3 split). (c) MAE values from five-fold cross-validation. (d) RMSE values from five-fold cross-validation (e) MAPE values from five-fold cross-validation.

Figures. S10 the converage of NSGA-II and NSGA-II-PMCP


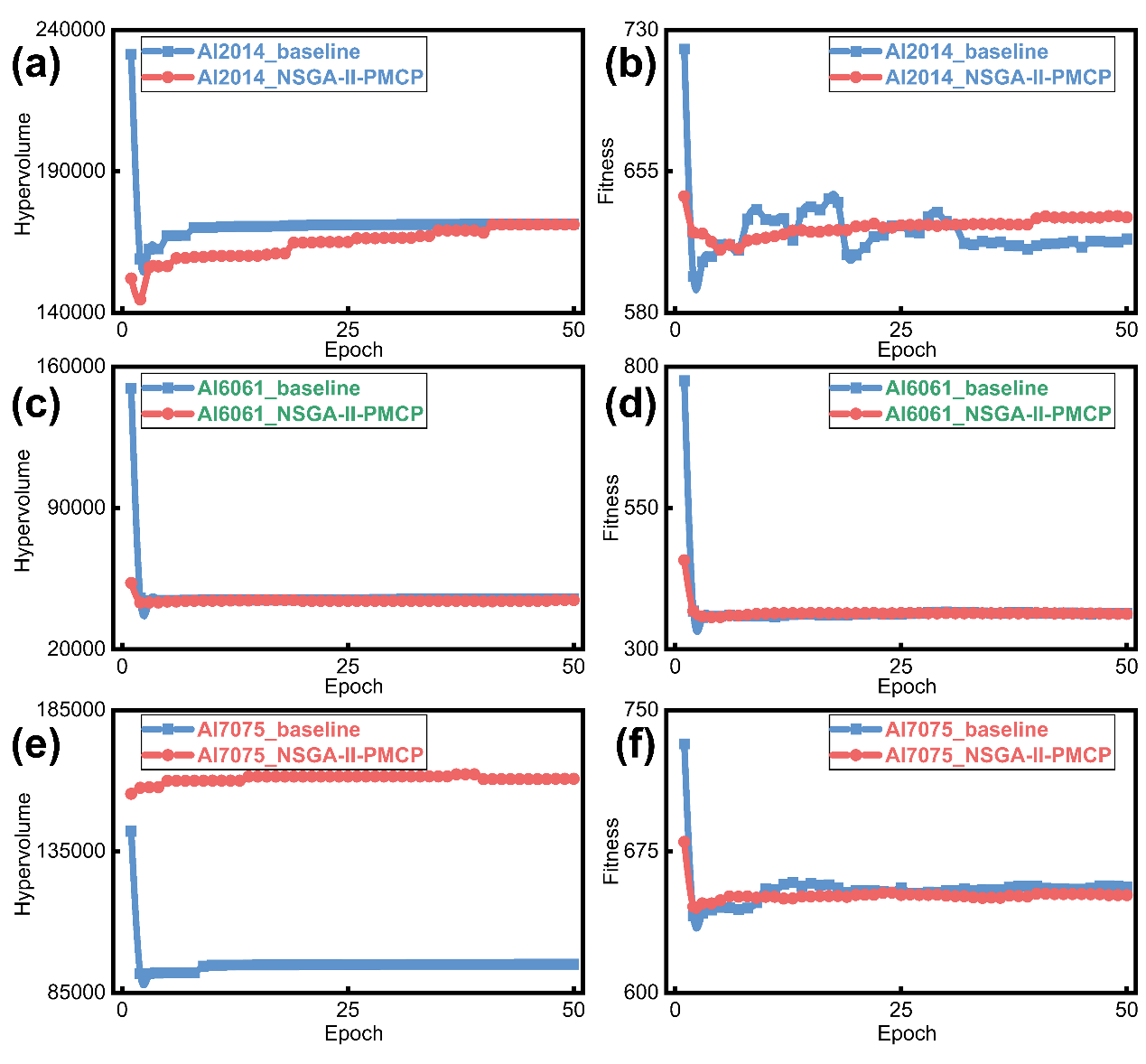


**Figures. S10.** The hypervolume and fitness of the Al2014-based PRAMCs, Al6061-based PRAMCs and Al7075-based PRAMCs.

Figures. S11 the comparison of optimization outcomes


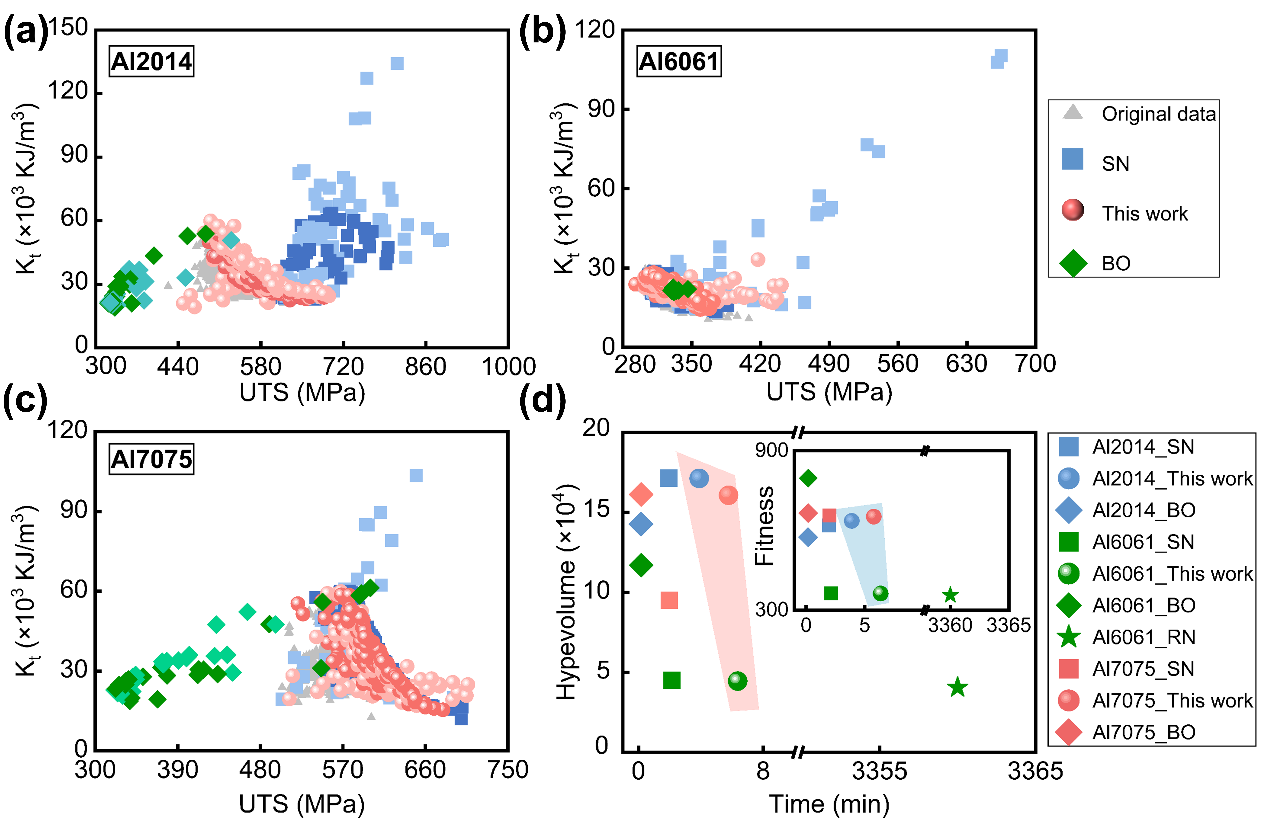


**Figures. S11.** Comparison of optimization outcomes for (a) Al2014-based PRAMCs, (b) Al6061-based PRAMCs, and (c) Al7075-based PRAMCs under four strategies including standard NSGA-II(SN), NSGA-II-PMCP (this work), Bayesian optimization (BO), and RFR-NSGA-II (RN), respectively. Colors denote distinct methods, while the color intensity from light to dark indicates the progression of iterations from early to late for each method. SN gradually converges from regions far from the original dataset toward the initial dataset, this work converges from below the original dataset toward the optimal dataset, and BO shows limited number improvement. (d) Comparisons of HV, fitness, and computational time across four methods. Colors are used to distinguish between different matrices. The light red shading highlights the performance of NSGA-II-PMCP in the HV versus computational time plot, while the light blue shading highlights its performance in the fitness versus computational time plot.

**Supplementary Tables**

Tables. S1 Simulation parameters- interface and reinforcement

| Material | Density (g/cm^3^) | Yang’s Modulus (GPa) | Poisson's ratio |
| --- | --- | --- | --- |
| SiC | 3.2 | 427 | 0.17 |
| Al_4_C_3_^[1]^ | 2.36 | 309 | 0.2 |
| Mg_2_Si^[2, 3]^ | 1.99 | 320 | 0.181 |
| Al_4_Si_3_^[4, 5]^ | 2.7 | 65.2 | 0.37 |

Tables. S2 Benchmark of the data (Simulation (Sim.), Reference (Ref.))

| Alloys | Volume fraction | SiC diameter | *E* | | *UTS* | | K_t_/elongation (%) | | Ref. |  |
| --- | --- | --- | --- | --- | --- | --- | --- | --- | --- | --- |
|  |  |  | True | Sim. | True | Sim. | True | Sim. |  |  |
| 2A02 | 0 | 0 | / | 68.14_-2_^+3^ | 558 | 560 | 14% | 13.9% | [6] | |
| 2A16 | 0 | 0 | / | 69.01 | 439.56 | 462 | - | - | [7] | |
| Al2016 | 0 | 0 | 72.9 | 70.1 | 481 | 484.97 | 14.5% | 15% | [8] | |
| Al2618 | 0 |  | / | 68.63 | 455.69 | 451.34 | 12% | 12.2% | [9] | |
| Al2014 | 0 | 0 | / | 66.61 | 462_-10_^+8^ | 473.17 | 9.8_-0.3_^+0.4^% | 10.2% | [10] | |
| Al2014  Random | 4% | 10 | / | 76.96±5 | 530_-14_^+9^ | 489.78±10 | 6.556 | 6.435 | [11] | |
|  | 4% | 10 |  | 76.96±5 | 512 ± 5 | 489.78±10 | 5.7 ± 0.6% | 6.435% | [12] | |
|  | 4% | 5 |  | 78.84±5 | 498 ± 6 | 502.70±10 | 5.3 ± 0.9% | 6.188% | [12] | |
| Al2024 | 0 | 0 | / | 67.85 | 660 | 660.82 | 10% | 10.72% | [13] | |
| Al6082 | 0 | 0 | / | 68.36 | 362.5 | 364.84 | / | - | [14] | |
| Al6061 | 0 | 0 | 68.9 | 65.16±6 | 305±10 | 302.61 | 11.8% | 12.37±0.5% | [15] | |
| Al6061  randomly distributed | 15% | 10 | - | 85 | 334 | 334 | - | - | [15] | |
|  | 15% | 20 | - | 91 | 335 | 334 | - | - | [15] | |
|  | 15% | 16 | - | 89 | 340 | 340 | - | - | [16] | |
| Al6061  randomly distributed spheres | 15% | 7.5 | - | 91 | 345 | 335 | - | - | [17] | |
| Al6061  network distributed polyhedra | 10% | 11 | 89 | 84 | 331 | 322 | 9.99 | 10.07 | [18] | |
| Al6061  micro/ Nano | Pure Al | - | 68.8 | 68 | 255 | 251 | 34.97 | 34.67 | [19] | |
|  | 1%nano+  9%micro | micro/nano | 95 | 90 | 309 | 281 | 18.7 | 18.54 | [19] | |
| Al7075 | 0 | 0 | 70.60 | 67.772 | 561.96 | 556.712 | 10.06% | 10.57% | [20] | |
|  | 5% | Micro | 70.69 | 72.03 | 555.69 | 551.726 | 3.49% | 5.51±1.5% | [20] | |

Tables. S3 SHAP Feature importance

| Feature | *UTS* | *K_t_* | *E* | SUM |
| --- | --- | --- | --- | --- |
| Fracture strain | 0.00297 | 0.00117 | 0.00041 | 0.00442 |
| Elastic modulus | 0.00058 | 0.00043 | 0.00038 | 0.00032 |
| A | 0.09736 | 0.14038 | 0.00702 | 0.24427 |
| B | 0.01171 | 0.00737 | 0.00125 | 0.01989 |
| n | 0.03030 | 0.01833 | 0.00337 | 0.05175 |
| C | 0.00652 | 0.00687 | 0.00105 | 0.00242 |
| Volume interface | 0.01410 | 0.00858 | 0.00146 | 0.02404 |
| Interface thickness | 0.01476 | 0.00888 | 0.00147 | 0.02499 |
| Al_4_C_3_ | 0.00814 | 0.00502 | 0.00089 | 0.01392 |
| Mg_2_Si | 0.00092 | 0.00043 | 0.00016 | 0.00140 |
| Al_4_Si_3_ | 0.00071 | 0.00023 | 0.00044 | 0.00100 |
| Configurations | 0.00203 | 0.00628 | 0.00621 | 0.01327 |
| Diameter | 0.00479 | 0.00915 | 0.00448 | 0.01244 |
| Volume Fraction | 0.01852 | 0.03722 | 0.04629 | 0.03020 |
| Varience | 0.00091 | 0.00177 | 0.00098 | 0.00244 |

Tables. S4 Static model regression parameters

| Model | Parameter | Abbreviation in code | Value |
| --- | --- | --- | --- |
| Linear Regression | Fit intercept | fit_intercept | True |
|  | Number of jobs | n_jobs | None |
| Decision Tree | Criterion | criterion | squared_error |
|  | Maximum depth | max_depth | None (unlimited) |
|  | Minimum samples per split | min_samples_split | 2 |
| Support Vector Machine | Kernel | kernel | rbf |
|  | Regularization parameter | C | 1.0 |
|  | Epsilon | epsilon | 0.1 |
| Gaussian Process | Kernel | kernel | RBF (length=1.0) × C=1 |
|  | Restarts optimizer | n_restarts_optimizer | 10 |
| K-Nearest Neighbors | Number of neighbors | n_neighbors | 5 |
|  | Weight function | weights | uniform |
| Neural Network | Hidden layer sizes | hidden_layer_sizes | (100, 50) |
|  | Activation | activation | relu |
|  | Solver | solver | adam |
|  | Max iterations | max_iter | 1000 |
| Random Forest | Number of trees | n_estimators | 100 |
|  | Maximum depth | max_depth | 100 |
|  | Minimum samples per split | min_samples_split | 5 |

Tables. S5 BPNN-CL hypeparameters

| Parameters | Hidden | Activation | Optimizer | Loss  Function |
| --- | --- | --- | --- | --- |
| Number | 128>64>32>16>8 | ReLU | Adam | MSE |

Tables. S6 BPNN-CL architecture

| Layer | Layer type | Output shape |
| --- | --- | --- |
| 1 | Input layer | (None,13) |
| 2 | Dense (ReLU) | (None, 128) |
| 3 | Dense (ReLU) | (None, 64) |
| 4 | Dense (ReLU) | (None, 32) |
| 5 | Dense (ReLU) | (None, 16) |
| 6 | Dense (ReLU) | (None, 8) |
| 7 | Dense (Linear) | (None, 3) |

Tables. S7 The Computational time and memory complexity

| Model | Inference time (ms) | Memory (KB) | Parameters |
| --- | --- | --- | --- |
| Linear Regression | 0.05106 | 1.5674 | 48 |
| Decision Tree | 0.0727 | 2.0039 | N/A (tree) |
| Support Vector Machine | 0.72927 | 19.7258 | N/A (#SV) |
| Gaussian Process | 0.51882 | 152.6166 | N/A (kernel) |
| K-Nearest Neighbors | 0.29764 | 11.6963 | N/A (lazy) |
| Random Forest | 5.58342 | 28.4018 | N/A (forest) |
| NN (5-layer) | 0.10363 | 2.3047 | 13983 |
| This work | 2.60531 | 18.3896 | 12819 |

Tables. S8 Comparison between BPNN-CL and references (Simulation (Sim.), Reference (Ref.))

| Matrix | Particle Configuration | Exp./sim. | *UTS* | | | *E* | | | Ref. |
| --- | --- | --- | --- | --- | --- | --- | --- | --- | --- |
|  |  |  | True | Pred. | Error | True | Pred. | Error |  |
| Al | Micro/nano | Exp. | 291.2 ±7.4 | 337.61 | 13.06% | 93.5 ±1.1 | 94.16 | 0.47% | [19] |
|  | Micro/nano | Exp. | 308.1 ±2.8 | 370.64 | 19.22% | 93.9 ±0.9 | 102.40 | 8.02% | [19] |
|  | Micro/nano | Exp. | 374.7 ±3.1 | 370.14 | 0.39% | 94.6 ±1.2 | 102.16 | 6.64% | [19] |
|  | Micro/nano | Exp. | 344.2 ±3.9 | 370.89 | 6.55% | 95.1 ±1.6 | 102.52 | 6.02% | [19] |
|  | Micro/nano | Exp. | 308.5 ±3.4 | 370.64 | 18.83% | 95.0 ±0.8 | 102.40 | 6.89% | [19] |
|  | Micro/nano | Exp. | 304.6 ±5.3 | 453.09 | 46.21% | 70.3 ±1.2 | 81.60 | 14.13% | [19] |
|  | Micro/nano | Exp. | 290.7 ±3.6 | 342.46 | 16.36% | 91.8 ±0.8 | 96.63 | 4.35% | [19] |
|  | Micro/nano | Exp. | 291.2 ±7.4 | 337.61 | 13.06% | 93.5 ±1.1 | 70.20 | 24.02% | [19] |
|  | Random | Exp. | 378.6 ±3.5 | 357.42 | 4.71% | 100.1 ±5.8 | 117.33 | 10.79% | [21] |
| X2080 | Random | Exp. | 574.359 | 544.01 | 5.28% | / | 99.93 | / | [22] |
| Al2024 | Random | Exp. | 352.192 | 372.24 | 5.69% | / | 88.57 | / | [23] |
|  | Random | Exp. | 401.792 | 387.46 | 3.57% | / | 102.13 | / | [24] |
|  | Random | Exp. | 433.812 | 452.65 | 4.34% | / | 108.51 | / | [24] |
| 2618 | Random | Sim. | 543.246 | 577.39 | 6.28% | / | 91.22 | / | [25] |
|  | Random | Sim. | 483.451 | 454.44 | 6.00% | / | 94.25 | / | [25] |
|  | Random | Sim. | 465.443 | 358.58 | 22.96% | / | 98.52 | / | [25] |
|  | Random | Exp. | 440.497 | 454.44 | 3.17% | / | 94.25 | / | [25] |
|  | Random | Exp. | 416.518 | 423.24 | 1.61% | / | 101.19 | / | [25] |
| A357 | Random | Exp. | 350 | 375.86 | 7.39% | 80.1 | 118.54 | 48.00% | [26] |
|  | Random | Exp. | 345 | 375.86 | 8.95% | 75 | 118.54 | 58.06% | [26] |
|  | Random | Exp. | 372 | 390.49 | 4.97% | 91.5 | 118.51 | 29.52% | [26] |
|  | Random | Exp. | 375 | 390.49 | 4.13% | 83.6 | 118.51 | 41.76% | [26] |
|  | Random | Exp. | 450 | 429.09 | 4.65% | 109.8 | 131.76 | 20.00% | [26] |
|  | Random | Exp. | 452 | 429.09 | 5.07% | 103.1 | 131.76 | 27.80% | [26] |
| AlSi7Mg | Random | Exp. | 502.94 | 547.30 | 8.82% | 94.53 ± 5.10 | 72.97 | 18.41% | [27] |
| A6061 | Random | Exp. | 424 | 415.77 | 1.94% | 90 | 90.68 | 0.75% | [28] |
|  | Random | Exp. | 449 | 453.86 | 1.08% | 99 | 96.17 | 2.86% | [28] |
|  | Random | Exp. | 405 | 408.87 | 0.96% | / | 92.86 | / | [29] |
|  | Network | Sim. | 358.185 | 319.02 | 10.93% | / | 85.89 | / | [30] |
|  | Uniform | Sim. | 341.545 | 319.67 | 6.40% | / | 85.06 | / | [30] |
|  | Network | Sim. | 330.647 | 324.08 | 1.99% | / | 92.89 | / | [31] |
| Al-Mg-Si | Random | Exp. | 364 ± 5 | 378.78 | 2.65% | / | 118.35 | / | [32] |
|  | Random | Exp | 403 ± 1 | 362.49 | 9.83% | / | 111.98 | / | [32] |
|  | Random | Exp. | 431 ± 3 | 385.94 | 9.83% | / | 92.00 | / | [32] |
| Al7075 | Random | Exp. | 437.1 ±9.2 | 418.51 | 2.19% | / | 103.36 | / | [33] |
|  | Random | Exp. | 583.5 ±1.8 | 608.85 | 4.02% | 95.5 ± 0.3 | 85.97 | 9.69% | [34] |
|  | Random | Exp. | 644.5 ±2.1 | 689.00 | 6.56% | 92.0 ± 0.3 | 87.88 | 4.16% | [34] |
| 7A04 | Random | Exp. | 586.574 | 618.55 | 5.45% | / | 83.96 | / | [35] |
|  | Random | Exp. | 568.6±1.3 | 614.31 | 7.79% | 84.2±0.7 | 72.86 | 12.74% | [36] |
|  | Random | Exp. | 581.4±3.5 | 599.74 | 2.54% | 97.4±4.5 | 87.81 | 5.48% | [36] |
|  | Random | Exp. | 623.6±5.2 | 660.83 | 5.09% | 113.3±0.8 | 102.36 | 9.01% | [36] |

Tables. S9 NSGA-II hypeparameters

| Parameters | Population Size | Maximum number of generations | Refer point |
| --- | --- | --- | --- |
| Al2014 | 50 | 50 | [700, 66] |
| Al6061 | 50 | 50 | [400,40] |
| Al7075 | 50 | 50 | [700,60] |

Tables. S10 NSGA-II-PMCP hypeparameters

| Parameters | Al2014 | Al6061 | Al7075 |
| --- | --- | --- | --- |
| pop_size | 50 | 50 | 50 |
| generations | 50 | 50 | 50 |
| Refer point | [700, 66] | [400,40] | [700,60] |
| crossover | SBX(prob=0.9, eta=15) | SBX(prob=0.9, eta=15) | SBX(prob=0.9, eta=15) |
| mutation | PM(eta=20) | PM(eta=20) | PM(eta=20) |

Tables. S11 the non-uniqueness solution

| Materials | *UTS* (MPa) | *K_t_*（×10^3^ KJ/m^3^） | Configurations | Diameter  (μm) | Volume Fraction (%) |
| --- | --- | --- | --- | --- | --- |
| SiCp/Al2014 | 601.3759 | 29.34607 | Micro/nano | 8 | 16 |
| SiCp/Al2014 | 612.326 | 30.29137 | Laminated | 12 | 17 |
| SiCp/Al2014 | 602.7839 | 31.70457 | uniform | 18 | 18 |
| SiCp/Al6061 | 358.9713 | 16.99571 | Cluster | 20 | 23 |
| SiCp/Al6061 | 357.8151 | 16.42371 | Micro/nano | 15 | 22 |
| SiCp/Al6061 | 357.3461 | 17.3521 | Laminated | 19 | 21 |
| SiCp/Al7075 | 645.6259 | 21.67513 | Micro/nano | 11 | 21 |
| SiCp/Al7075 | 641.9195 | 22.24731 | Micro/nano | 12 | 20 |
| SiCp/Al7075 | 646.4631 | 21.05184 | Network | 17 | 23 |

**Principles supplement**

Principles. S1: The reaction principle of Al_4_C_3_, Mg_2_Si and Al_4_Si_3_

The wettability between SiC particles and molten Al alloy is relatively poor within the temperature range of 700°C to 900°C. As the temperature increases, interfacial wettability improves; however, excessive temperatures promote the formation of brittle and hydrolyzable Al₄C₃ phases. Considering the melting point range of aluminum alloys, if the sintering temperature exceeds this range, the Al alloy transitions into a molten state. Upon contact with the molten Al alloy, SiC particles begin to dissolve. Once the interfacial C concentration reaches saturation, continued dissolution of SiC leads to the precipitation of Al₄C₃ in blocky and needle-like morphologies at the interface.^[37, 38]^

| $3SiC+4Al\to Al_{4}C_{3}+3Al$ | (1-1) |
| --- | --- |

Al₄C₃ and Si are brittle phases that are prone to microcrack formation under external loads, thereby deteriorating the mechanical properties of the composites. Additionally, due to the hydrophilic nature of Al₄C₃, the decomposition reaction described in equation (1-2) readily occurs in humid environments, leading to composite degradation. Moreover, the presence of Al₄C₃ at the interface acts as a thermal barrier, reducing the thermal conductivity of the composites.

| $Al_{4}C_{3}+18H_{2}O\to4Al(OH)_{3}+3CO_{2}+12H_{2}$ | (1-2) |
| --- | --- |

In addition to the SiC and Al phases in the matrix, weak amount of the Mg₂Si and MgAl₂O₄ phases are observed in the composites.^[39]^ This indicates that reactions described by equations (1-3)– (1-4) may have occurred during the sintering process.

| $2SiO_{2}+Mg+2Al\to MgAl_{2}O_{4}+2Si$ | (1-3) |
| --- | --- |
| $Si+2Mg\to Mg_{2}Si$ | (1-4) |

The introduction of Al into SiC_f_/SiC composites facilitates the formation of β-SiC and promotes grain growth.^[40]^ When the aluminum content exceeds 10%, a significant amount of Al₄Si₃ phase begins to form.

Principles. S2 Johnson-Cook model

The Johnson-Cook plasticity model is a specialized form of the Mises plasticity model, incorporating analytical expressions for the hardening law and strain rate dependence. It is particularly suitable for simulating high-strain-rate deformation in various materials, including most metals.

**2.1 Johnson-Cook hardening**

Johnson-Cook hardening is a specific type of isotropic hardening in which the static yield stress $\sigma^{0}$ is expressed as:

| $\sigma^{0}=[A+B(\bar{\varepsilon}^{pl})^{n}](1-\hat{\theta}^{m})$ | (2-1) |
| --- | --- |

where $\bar{\varepsilon}^{pl}$ is the equivalent plastic strain and *A*, *B*, *n* and *m* are material parameters measured at or below the transition temperature, $\theta_{transition}$, $\hat{\theta}$ is the nondimensional temperature defined as:

| $\hat{\theta}\left\{\begin{matrix} 0 \\ (\theta-\theta_{transition})/(\theta_{melt}-\theta_{transition}) \\ 1 \end{matrix}\right.\begin{matrix} for \\ for \\ for \end{matrix}\begin{matrix} \theta<\theta_{transition} \\ \theta_{transition}<\theta<\theta_{melt} \\ \theta_{melt}<\theta\end{matrix}$ | (2-2) |
| --- | --- |

where *θ* is the current temperature, *θ_melt_* is the melting temperature, and *θ_transition_* is the transition temperature, defined as the temperature at or below which the yield stress exhibits no temperature dependence. The material parameters *A*, *B*, *n*, and *m* must be determined at or below the transition temperature to ensure accurate characterization of the material's plastic behavior.

When *θ≥θ_melt_*, the material undergoes melting and behaves like a fluid, resulting in no shear resistance (σ_0_=0). At this stage, the material's hardening memory is erased by resetting the equivalent plastic strain to zero. Additionally, if backstresses are defined in the model, they will also be set to zero to reflect the loss of material strength and rigidity.

**2.2 Johnson-Cook strain rate dependence**

Johnson-Cook strain rate dependence assumes that

|  | (2-3) |
| --- | --- |

and

|  | (2-4) |
| --- | --- |

Where: $\bar{\sigma}$ is the yield stress at nonzero strain rate; is the the equivalent plastic strain rate; and *C* are material parameters measured at or below the transition temperature, *θ_transition_*; $\sigma_{0}(\bar{\varepsilon}^{pl},\theta)$ is the static yield stress; and is the ratio of the yield stress at nonzero strain rate to the static yield stress (so that =1.0). The yield stress is, therefore, expressed as:

|  | (2-5) |
| --- | --- |

Principles. S3 FEA principles, Mesh convergence, Toughness integral, influence of specimen-level necking and fracture, and anisotropy analysis ^[41]^

**3.1 FEA principles**

The mechanical behavior, including the stress-strain response of the analyzed representative volume elements (RVEs), is simulated using ABAQUS 2022. To ensure appropriate boundary conditions, the YX surface of the 3D RVE is fully constrained in all degrees of freedom, while the opposite XY surface undergoes a gradual displacement along its normal direction to simulate loading conditions. This study considers different series of aluminum alloys, three types of interface products, and various reinforcement parameters, including configurations, volume fractions, diameters, and diameter variations. The detailed parameters are provided above.

During the deformation process, fracture initiation and propagation are attributed to the progressive loss of load-bearing capacity, driven by material stiffness degradation. To accurately capture this behavior, a fracture criterion is incorporated into the simulations. Once damage occurs in the FEA model, the mechanical response is predominantly governed by the evolution of stiffness degradation, which is quantitatively represented through a predefined damage model.

| $\sigma=(1-D)\overline{\sigma}$ | (3-1) |
| --- | --- |

**Where *D* and $\sigma$ represent the overall damage variable and the degraded stress tensor, respectively. $\overline{\sigma}$ denotes the stress tensor without damage. When all cross-sectional points of an element lose their load-bearing capacity, the element is removed from the simulation.**

Once material damage occurs, the conventional stress-strain relationship can no longer accurately describe the mechanical behavior of the examined system. Continued reliance on this relationship leads to pronounced mesh dependency due to strain localization, resulting in reduced energy dissipation as the mesh is refined. To address this issue, an alternative approach is required to capture the strain-softening behavior observed in the stress-strain response curve. Notably, the introduction of fracture energy by Hillerborg et al. effectively mitigates mesh dependency by defining a stress-displacement relationship after damage initiation. Consequently, the post-damage softening response can be characterized using the well-defined fracture energy *G_f_*, which describes energy dissipation per unit crack area, rather than relying solely on the stress-strain curve.

| $G_{f}=\int_{\overline{\varepsilon}_{0}^{pl}}^{\overline{\varepsilon}_{f}^{pl}} L\sigma_{y}d\overline{\varepsilon}^{pl}=\int_{0}^{\overline{u}_{f}^{pl}} \sigma_{y}d\overline{u}^{pl}$ | (3-2) |
| --- | --- |

where *L* is the characteristic length, $\sigma_{y}$ is yield stress, and $\overline{u}^{pl}$is the equivalent plastic displacement. $\overline{\varepsilon}_{0}^{pl}$and $\overline{\varepsilon}_{f}^{pl}$are the equivalent plastic strain at the onset of damage and the equivalent plastic strain at failure respectively. In Eq. 3-2, when the damage starts, $\overline{u}^{pl}$satisfies the following equation.

| $\overline{u}^{pl}=L\overline{\varepsilon}^{pl}$ | (3-3) |
| --- | --- |

During the stretching process, it is noteworthy that the interface between the reinforcement and the matrix may undergo separation. However, recent simulation studies have shown that numerical results obtained for a strong interface exhibit a closer correlation with experimental data compared to those for weak interfaces. Given that the primary objective of this work is to optimize structural parameters to enhance both strength and toughness in HSMMCs, weak interfaces with bonding separation are not considered. Instead, the simulations focus solely on strong interfaces. Based on this framework, the stress-strain curves of the examined HSMMCs are recorded, along with key mechanical properties extracted from these curves, including elastic modulus, ultimate tensile strength and toughness.

**3.2 Mesh convergence**

In the article, the RVE size is set to 75 μm, while the mesh size ranges from 1.7 to 1.8 μm. To quantify the sensitivity of both mesh and RVE size, we define the mesh coefficient **(A)** as the ratio of RVE size to mesh size.

| $\xi=\frac{RVE\_size}{mesh\_size}$ | (3-4) |
| --- | --- |

We sequentially set the mesh coefficient **(A)** for the three examined configurations—random, network, and cluster distributions—to 7.5, 10, 15, 25, 30, 40, 42.5, 45, 47.5, and 50. The corresponding parameters and computational time are summarized in Table 1.

Table 1 Computational resources required under different mesh coefficients

| mesh coefficient ($\xi$) | mesh size | Time (min) |
| --- | --- | --- |
| 7.5 | 10 | 6 |
| 10 | 7.5 | 8 |
| 15 | 5 | 11 |
| 25 | 3 | 12 |
| 30 | 2.5 | 13 |
| 40 | 1.88 | 15 |
| 42.5 | 1.76 | 18 |
| 45 | 1.67 | 25 |

* The above data are the average values of network configurations

We sequentially set the mesh coefficient ($\xi$) from 7.5 to 50, and the corresponding configurations, stress-strain curves, and toughness-ultimate tensile strength relationships are presented in the following figures. As shown in (b), (e), and (h), for uniformly distributed polyhedral structures, an increase in the mesh coefficient (i.e., a decrease in mesh size) leads to a gradual reduction in both the peak stress of the stress-strain curve and the area under the curve. However, when the mesh coefficient increases beyond 40, the stress-strain curve remains nearly unchanged. Likewise, as observed in (c), (f), and (i), the variations in strength and toughness become negligible when $\xi$ reaches 40–50. Therefore, mesh convergence is considered achieved at $\xi$ = 40.


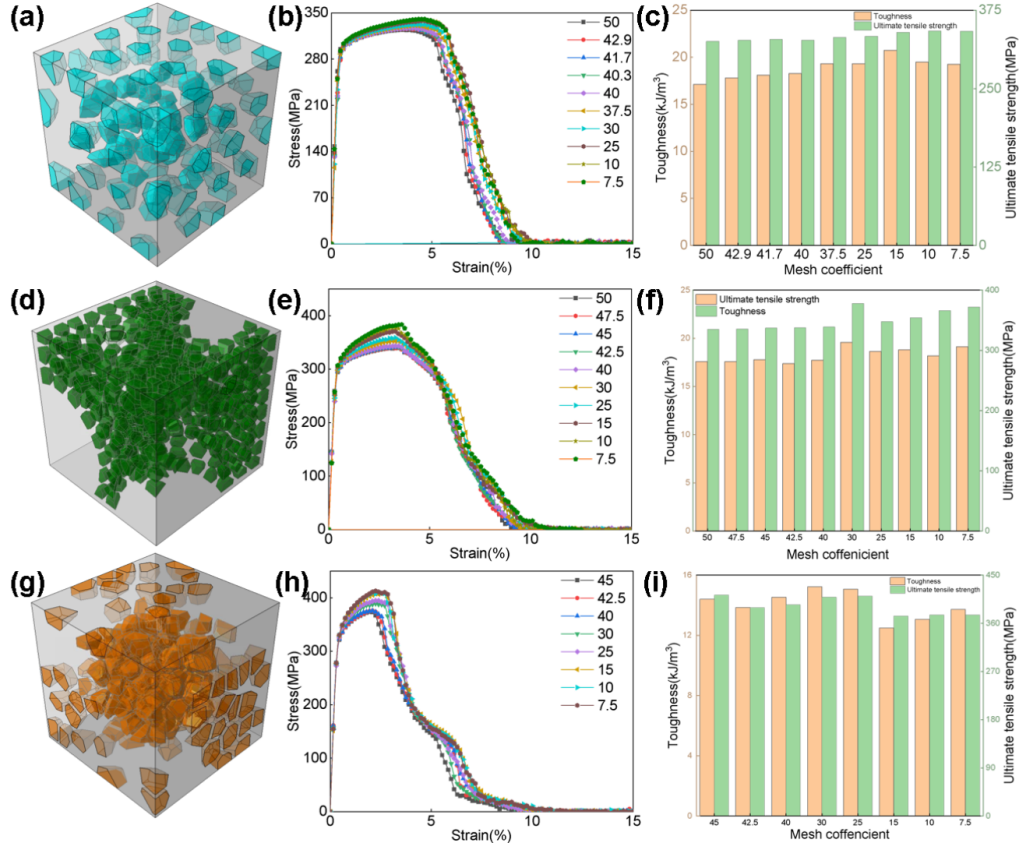


Figure 1. Comparison of stress-strain curves and strength-toughness under different mesh coefficients. (a), (d), and (g) random, network and cluster distributions. (b), (e), and (h) Stress-strain curves. (c), (f), and (i) strength-toughness comparison under different mesh coefficients

In summary, when the mesh coefficient reaches 40, the calculation accuracy stabilizes, and the overall trend of the curve remains unchanged with further increases in the mesh coefficient. As shown in Table 1, the computing time is approximately 15 minutes when the mesh coefficient is 40, whereas it doubles to 30 minutes when the mesh coefficient reaches 50. Considering the trade-off between computational efficiency and accuracy, we determine that the mesh coefficient should be greater than 40. In this study, the defined mesh coefficients range from 41.7 μm to 44.1 μm, which effectively ensures both convergence and computational efficiency.

**3.3 Toughness integral**

When MMC fails, the material is prone to fluctuations in the later stage of calculation. Therefore, this work defined that the calculation stops when the material stress reaches certain threshold. At this time, the area enclosed by the stress-strain curve is the material toughness, which is solved using the trapezoidal rule. Divide the integration interval [a, b] into *n* equal parts, each with a length of $h=\frac{(b-a)}{n}$. Then, take the function values corresponding to the two endpoints (a and b) in each subinterval as the height at both ends of the interval, and multiply them with h to obtain the trapezoidal area within the subinterval.

| $\int_{a}^{b} f(x)dx\approx\frac{h}{2}[f(a)+2f(x_{1})+\cdots+2f(x_{n-1})+f(b)]$ | (4-5) |
| --- | --- |

where $x_{1},x_{2},x_{3},\cdots,x_{n-1}$ is the right endpoint of the *n-1* subinterval obtained by dividing the interval [a, b] equally.


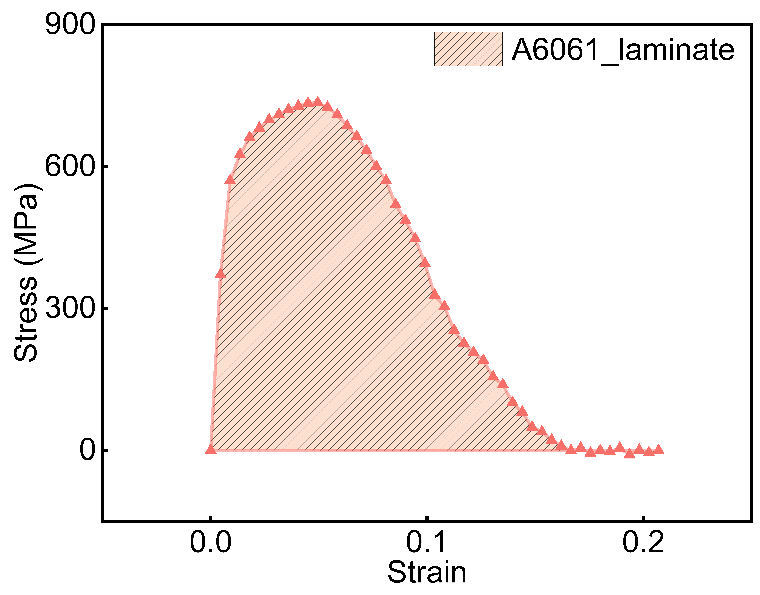


Figure 2. Integral figure

**3.4 Influence of specimen-level necking and fracture**

The experimental tensile stress–strain response of particle-reinforced aluminum matrix composites is affected not only by the intrinsic constitutive behavior of the matrix and reinforcement, but also by specimen-level structural instability, including strain localization, necking, and final fracture. Therefore, the macroscopic experimental engineering stress–strain curve and the homogenized response extracted from the representative microstructural finite-element model should not be regarded as fully equivalent over the entire deformation range.

In the present work, the simulated response represents the effective response of a representative microstructure under a prescribed uniaxial tensile boundary condition. It captures the effects of particle diameter, volume fraction, spatial configuration, and matrix constitutive behavior, but does not explicitly reproduce the complete macroscopic necking process of a tensile specimen. For this reason, *UTS* was selected as the primary strength metric for comparison, because it is less affected by post-necking deformation than fracture strain or total fracture energy. By contrast, the toughness-related metric *K_t_* is obtained from the area under the stress–strain curve and is therefore more sensitive to damage evolution, strain localization, and post-peak deformation.

The fracture behavior of the target metal matrix composites is not assumed to be universally ductile. It depends on SiC volume fraction, particle size, particle clustering, reinforcement architecture, and interfacial integrity. At relatively low or moderate particle volume fractions with dispersed particles and continuous interfaces, the composite may retain a ductile or mixed ductile fracture mode, with matrix plastic deformation and local particle-related damage. However, when the volume fraction is high, when particles form connected or clustered structures, or when interfacial defects are present, crack initiation and propagation may become more localized, and the fracture response may shift toward a more brittle or mixed brittle–ductile mode.

**3.5 Anisotropy analysis**

**(1) Evaluation of directional responses**

To quantitatively evaluate the anisotropy of the representative microstructural models used in this work, 24 models were selected from the four reinforcement configurations considered in this study, namely clustered, networked, uniform, and laminated structures. For each selected model, uniaxial tensile simulations were performed independently along the X, Y, and Z directions. The corresponding stress–strain curves are shown in Figure 3. For the 24 models examined, the stress–strain responses obtained along the three orthogonal loading directions are generally consistent in the elastic regime and over most of the plastic deformation stage. In many cases, the curves for the same model under different loading directions nearly overlap. This indicates that the randomly oriented polyhedral SiC reinforcements generated by Neper lead to a statistically near-isotropic response at the model-ensemble level, although individual polyhedral particles are geometrically anisotropic.

For the clustered and uniform configurations (Figure 3 (a) and (c)), the stress–strain curves along the X, Y, and Z directions are almost indistinguishable over the entire deformation range, including the hardening stage and the onset of fracture. This behavior is consistent with the spatial distribution of reinforcements in these two configurations, where no preferred particle alignment is introduced. As a result, none of the three loading directions experiences a systematically different reinforcement arrangement. The same tendency is observed for different particle diameters and volume fractions, ranging from 11 to 12 μm and from 5% to 12%, respectively. This suggests that the observed isotropy is not caused by a single microstructural realization, but is a stable feature of the model generation procedure.

For the networked and laminated configurations (Figure 3 (b), (d)), some geometric anisotropy can be observed from the three-dimensional reinforcement arrangements, as these structures contain directional features by design. Even so, the stress–strain curves along the X, Y, and Z directions remain close in terms of elastic modulus and *UTS*. Noticeable directional differences appear mainly after the peak stress, during the fracture stage. This result suggests that the elastic modulus and peak strength are governed primarily by the SiC volume fraction, particle size, and matrix response, whereas the reinforcement topology has a stronger influence on damage evolution and fracture-path selection. The moderate directional variation near fracture is therefore physically reasonable, as damage initiation and crack propagation are more sensitive to the local topology of the microstructure.


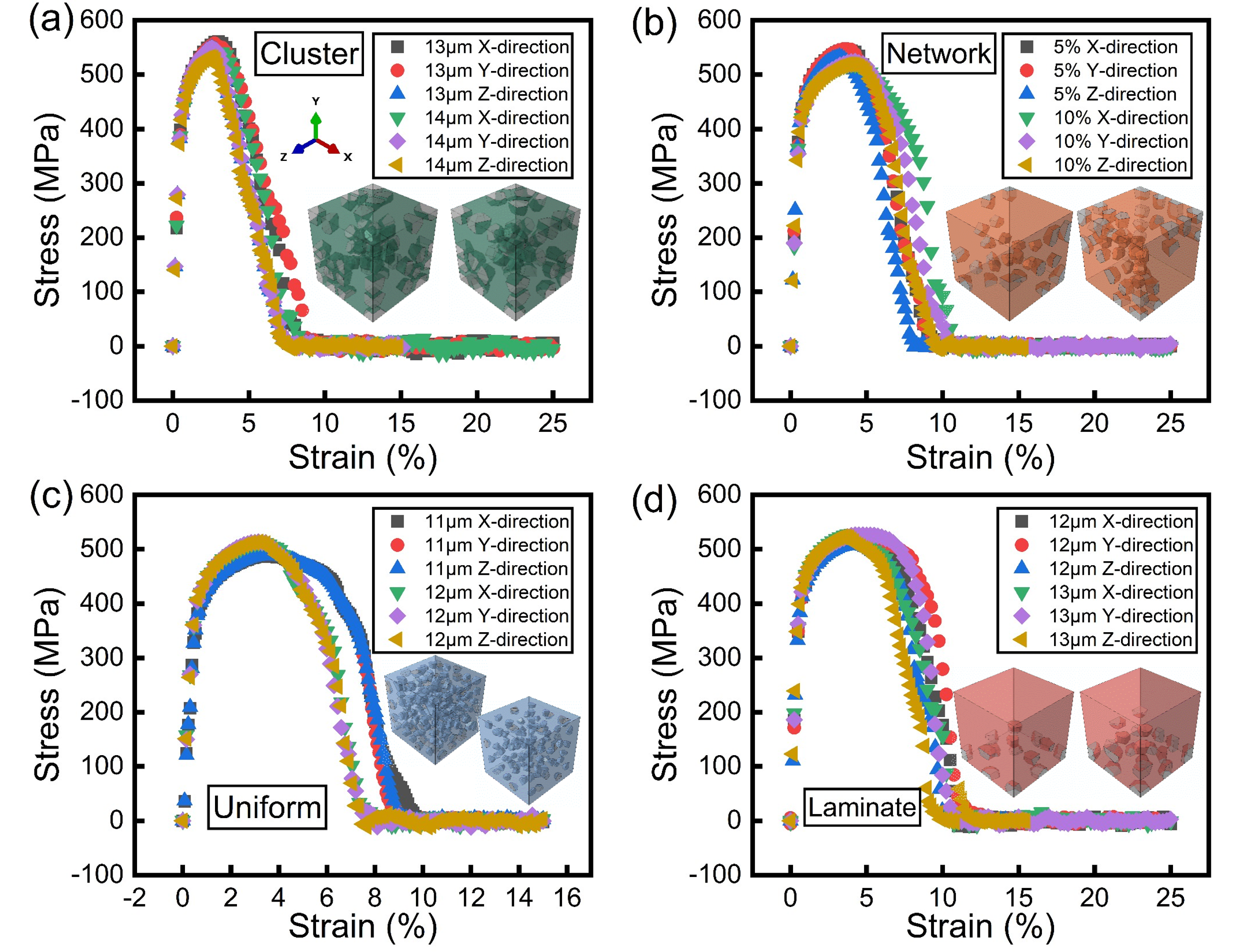


Figure 3. Directional tensile responses of representative microstructural models. (a) – (d), Stress–strain curves of Al2014-based PRAMCs under uniaxial tension applied along the X, Y and Z directions. (a), Clustered configuration with particle diameters of 13 μm and 14 μm at volume fractions of 15.5% and 16.5%, respectively. (b), Networked configuration with 12 μm particles at volume fractions of 5% and 10%. (c), Uniform configuration with particle diameters of 11 μm and 12 μm at volume fractions of 5% and 12%, respectively. (d), Laminated configuration with particle diameters of 12 μm and 13 μm at volume fractions of 3% and 5%, respectively.

Overall, the directional loading results support the use of a statistically near-isotropic assumption in the present framework for predicting elastic modulus and strength-related properties. At the same time, the residual directional dependence observed during post-peak fracture is acknowledged as a source of uncertainty and will be examined more systematically in future work.

**(2) Role of geometric descriptors related to particle shape**

The descriptor set used in this work was determined after examining the relative influence of particle geometry on the stress–strain response of HSMMCs. The considered geometric factors included particle shape, orientation, diameter, and volume fraction. This preliminary analysis was used to assess whether orientation- or shape-related descriptors should be included in the final feature set.

As shown in Figure 4 (a), stress–strain curves were calculated for representative microstructural models containing either polyhedral or spherical SiC particles. For the polyhedral particles, three prescribed orientations, 0°, 30°, and 60°, were considered. The particle diameter and volume fraction were fixed at 10 μm and 10%, respectively. The resulting curves show only minor differences among the tested particle shapes and orientations. This indicates that, within the parameter range considered here, particle orientation and the associated geometric anisotropy do not play a dominant role in the tensile response of the HSMMCs. Therefore, orientation-related descriptors were not included in the final descriptor set. This choice was made based on the numerical comparison rather than as an arbitrary simplification. By contrast, Figure 4 (b) shows that changing the particle diameter from 6 to 18 μm at a fixed volume fraction of 10% leads to a clear change in the stress–strain response. Figure 4 (c) further shows that the SiC volume fraction has a pronounced influence on both strength and fracture behavior. These results suggest that particle size and volume fraction are the main geometric factors controlling the mechanical response in the present system.


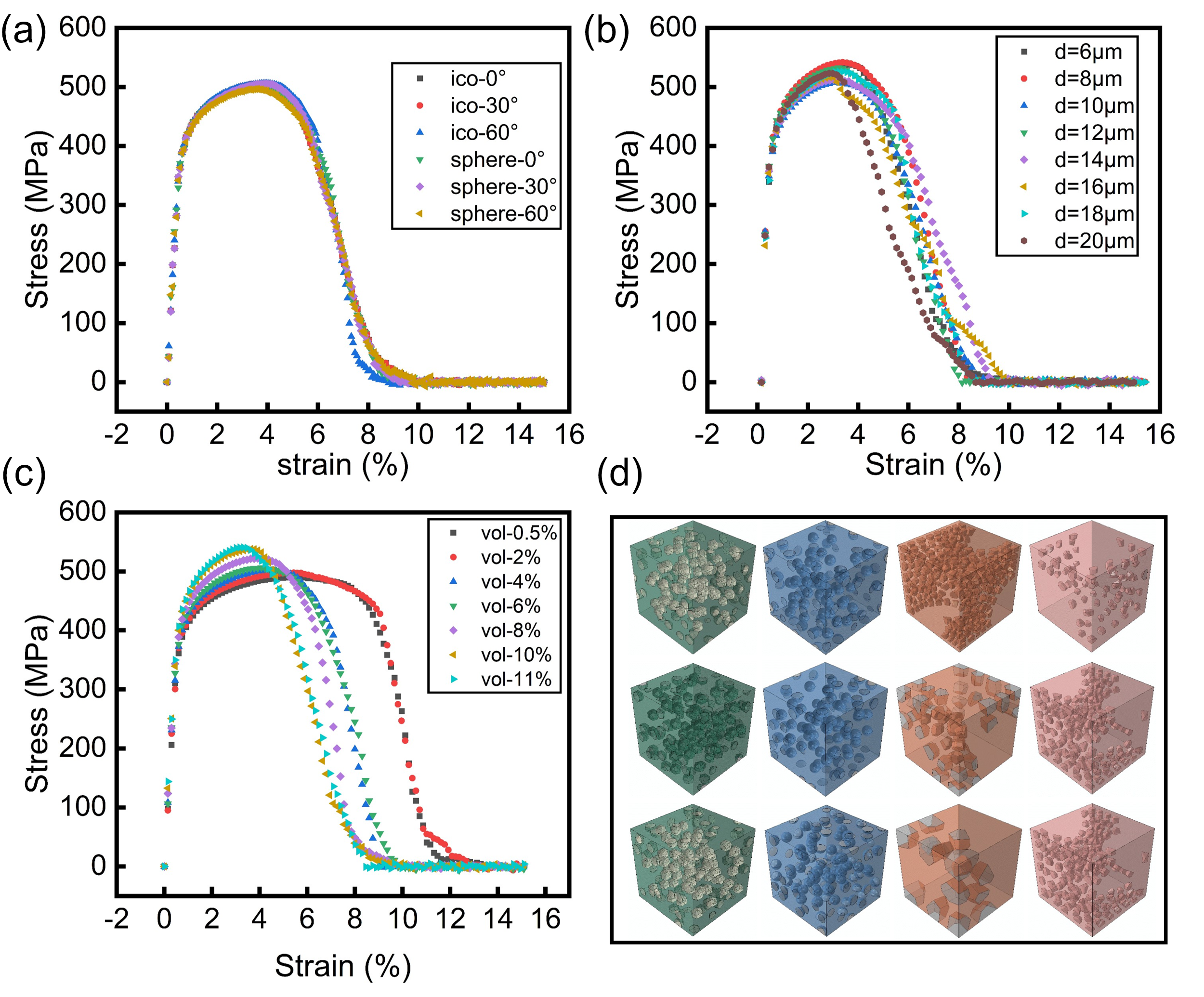


Figure 4**.** Effect of particle geometry on the tensile response of HSMMCs. (a)–(c), Stress–strain curves obtained from uniaxial tensile simulations with different particle geometries. (a) Comparison between polyhedral and near-spherical SiC particles with prescribed orientations of 0°, 30° and 60°, at a fixed particle diameter of 10 μm and volume fraction of 10%. (b) Effect of particle diameter for polyhedral SiC particles with diameters of 6, 12 and 18 μm at a fixed volume fraction of 10%. (c) Effect of SiC volume fraction in the network configuration at a fixed particle diameter of 10 μm, with volume fractions of 2%, 6% and 10%. (d) Representative three-dimensional microstructures corresponding to the parametric studies in (a) – (c), including particle orientation, particle shape, particle diameter and volume fraction variations.

Principles. S4 Mantel Test

**4.1 Mantel Test**

The Mantel test is used to assess the correlation between two distance matrices. It computes a correlation coefficient *r* that measures the degree of similarity between the two matrices. Given two matrices *A* and *B*, the Mantel correlation coefficient *r* is calculated using the following formula:

| $r=\frac{\sum_{i,j} (A_{ij}-\overline{A})(B_{ij}-\overline{B})}{\sqrt{\sum_{i,j} (A_{ij}-\overline{A})^{2}(B_{ij}-\overline{B})^{2}}}$ | (4-1) |
| --- | --- |

Where *A_ij_* and *B_ij_* are the elements of matrices of *A* and *B.* $\overline{A}$and $\overline{B}$ are the mean value of matrices *A* and *B*, respectively. The coefficient r is computed by comparing the pairwise distance in matrix *A* with those matrix *B*.

r<0.2: Weak correlation

0.2≤r<0.4: Moderate correction

R≥0.4：Strong correlation

**4.2 Categorization of the P-value**

The p-value is another result from the Mantel test, which indicates the statistical significance of the correlation. The p-value is categorized as follows:

p<0.01: Highly significant

0.01≤p<0.05: Moderately significant

p≥0.05: Not significant

Principles. S5 Random forest regression

The model is trained using Random Forest Regression (RFR), which consists of multiple decision trees and leverages ensemble learning to improve prediction accuracy.


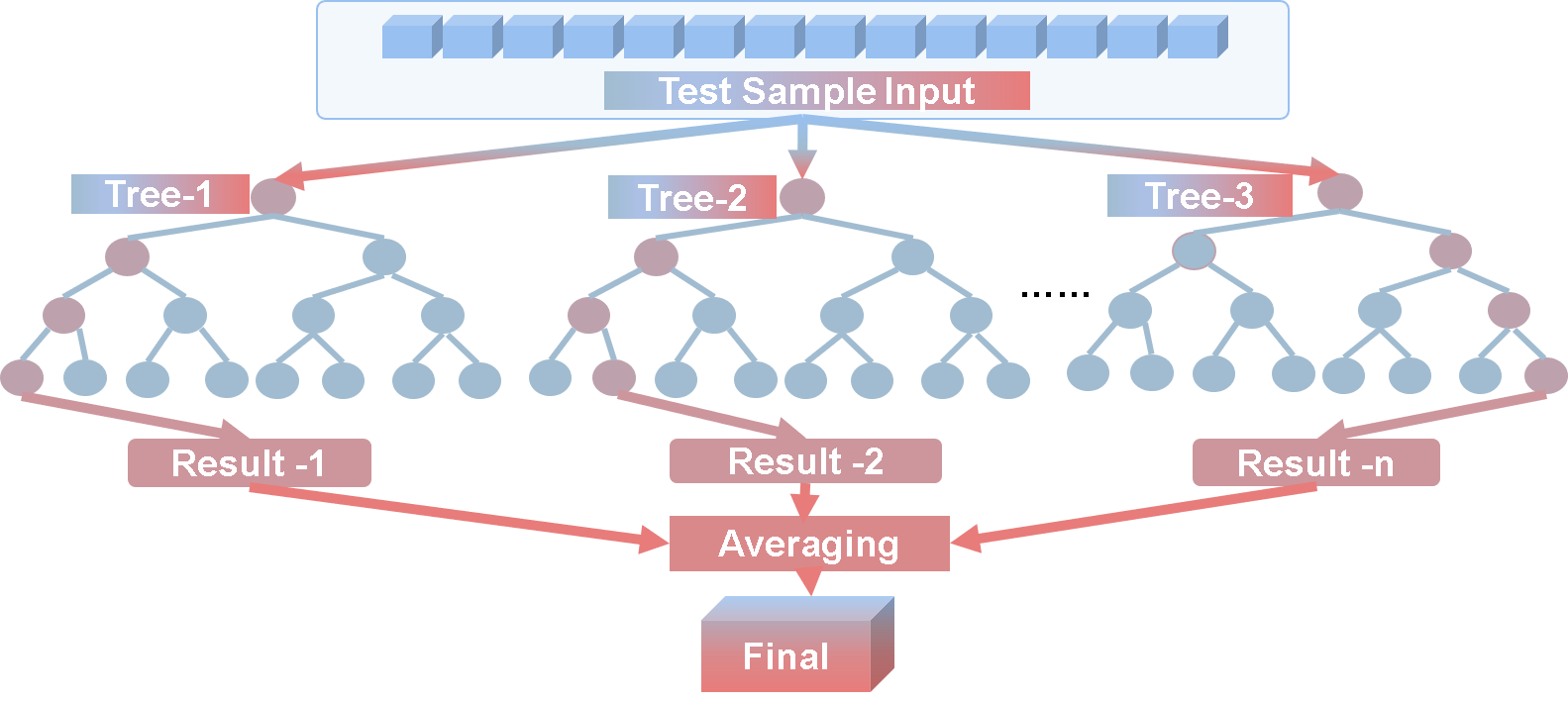


Figure 5. Random forest regression

**5.1 Prediction of a Single Decision Tree**

The basic prediction formula for a decision tree is:

| $y=f(x)=\sum_{i=1}^{N} w_{i}\cdot x_{i}+b$ | (5-1) |
| --- | --- |

Where *N* is the number of input features; $x_{i}$ is input variable; $w_{i}$is feature weight; $b$ is bias term.

**5.2 Prediction of Random Forest**

The final prediction of a random forest is the average prediction of multiple decision trees, given by:

| $\hat{y}=\frac{1}{T}\sum_{t=1}^{T} f_{t}(x)$ | (5-2) |
| --- | --- |

Where *T* is the number of decision trees. *f_t_(x)* is the prediction of the *t-th* decision tree.

**5.3** **Evaluation parameters**

1. Root Mean Square Error (RMSE)

| $RMSE=\sqrt{\frac{1}{n}\sum_{i=1}^{n} (y_{i}-\hat{y}_{i})^{2}}$ | (5-3) |
| --- | --- |

1. Mean Absolute Error (MAE)

| $MAE=\frac{1}{n}\sum_{i=1}^{n} \left\vert y_{i}-\hat{y}_{i}\right\vert$ | (5-4) |
| --- | --- |

1. Coefficient of Determination (R^2^)

| $R^{2}=1-\frac{\sum(y_{i}-\hat{y}_{i})^{2}}{\sum(y_{i}-\bar{y}_{i})^{2}}$ | (5-5) |
| --- | --- |

1. Mean Absolute Percentage Error (MAPE)

| $MAPE=\frac{1}{N}\sum\left\vert\frac{y_{i}-\hat{y}_{i}}{y_{i}}\right\vert\times100\%$ | (5-6) |
| --- | --- |

Principles. S6 SHAP Analysis

To interpret the model's predictions, the SHapley Additive exPlanations (SHAP) method is employed.

**6.1 SHAP Value Computation**

The SHAP values are calculated using the TreeExplainer method, based on the concept of Shapley values from cooperative game theory. The SHAP value for feature *j* in an individual prediction is defined as:

|  | (6-1) |
| --- | --- |

Where *f(x)* represents the model’s predictions for given input *X*, and E[*f(X)*] is the expectation of model output over all samples.

|  | (6-2) |
| --- | --- |

Where corresponds to the SHAP value of feature j for target (*UTS*, *K_t_*, or *E*) and n corresponds to the target nums.

**6.2 Feature Importance Evaluation for Each Target (*UTS*, *K_t_*, and *E*)**

The absolute mean SHAP value for each feature across all samples is computed as:

|  | (6-3) |
| --- | --- |

Where *N* is the number of test samples and denotes the SHAP value of feature *j* for sample *i*.

The global feature importance for the combined objective is obtained by averaging the absolute SHAP values:

|  | (6-4) |
| --- | --- |

**6.3 Hierarchical Clustering Analysis**

To assess feature relationships, hierarchical clustering is applied using single linkage clustering, defined as:

| $D(i,j)=\min_{x\in C_{i},y\in C_{j}} d(x,y)$ | (6-5) |
| --- | --- |

Where *C_i_* and *C_j_* represent different feature clusters, and *d(x, y)* is the Euclidean distance between feature values. Hierarchical clustering is performed on the combined SHAP values using single linkage clustering. This step helps identify feature groups that contribute similarly to the overall performance of *UTS*, *K_t_*, and *E*.

Principles. S7 Back Propagation Neural Network

The Back Propagation Neural Network (BPNN) is a feedforward neural network trained using backpropagation. In our case, it consists of an input layer, one or more hidden layers, and an output layer. Each layer performs the following computations:

**7.1 Forward propagation**

(1) Linear Transformation

| $Z^{(l)}=W^{(l)}A^{(l-1)}+b^{(l)}$ | (7-1) |
| --- | --- |

Where $W^{(l)}$ is the weight matrix of layer *l*, $A^{(l-1)}$ is the output of previous layer or input data for the first hidden layer and $b^{(l)}$ is the bias vector.

(2) Activation Function:

The activation function introduces non-linearity into the model. We use ReLU (Rectified Linear Unit):

| $A^{(l)}=f(Z^{(l)})$ | (7-2) |
| --- | --- |

Where:

| $f(Z)=\max(0,Z)$ | (7-3) |
| --- | --- |

This ensures that only positive values pass forward, preventing issues like the vanishing gradient problem.

Since we are solving a regression problem (predicting *UTS*, *K_t_*, and *E*), the output layer does not use any activation function:

| $Ypred=W^{(L)}A^{(L-1)}+b^{(L)}$ | (7-4) |
| --- | --- |

- 1. **Backpropagation (Error Calculation and Weight Updates)**

1. **Loss function**

| $L=MSE=\frac{1}{n}\sum_{i=1}^{n} (y_{i}-\hat{y}_{i})^{2}$ | (7-5) |
| --- | --- |

1. **Gradient Calculation for the Output Layer:**

The gradient of the loss with respect to the output layer’s activation is:

| $\delta^{(L)}=\frac{\partial L}{\partial Z^{(L)}}=(\hat{y}_{i}-y_{i})$ | (7-6) |
| --- | --- |

This error term will be propagated backward to adjust the weights.

1. **Error Backpropagation to Hidden Layers:**

Using the chain rule, we compute the gradient for hidden layers:

| $\delta^{(l)}=(W^{(l+1)}\delta^{(l+1)})\cdot f'(Z^{(l)})$ | (7-7) |
| --- | --- |

Where $f'(Z^{(l)})$ is the derivative of the ReLU activation function:

| $f'(Z)=\left\{\begin{matrix} 1,Z>0 \\ 0,Z>0 \end{matrix}\right.$ | (7-8) |
| --- | --- |

This allows errors from the output layer to propagate backward.

1. **Gradient Calculation for Weights and Biases:**
   The weight and bias updates are computed as:

| $\begin{matrix} \frac{\partial L}{\partial W^{(l)}}=\frac{1}{m}\sum_{i=1}^{m} \delta^{(l)}A^{(l-1)} \\ \frac{\partial L}{\partial b^{(l)}}=\frac{1}{m}\sum_{i=1}^{m} \delta^{(l)} \end{matrix}$ | (7-9) |
| --- | --- |

1. **Gradient Descent and Adam Optimization**

Instead of simple stochastic gradient descent (SGD), we use the Adaptive Moment Estimation (Adam) optimizer, which combines the benefits of momentum and adaptive learning rates.

1. **Momentum Update**:

| $\begin{matrix} m_{t}=\beta_{1}m_{t-1}+\left(1-\beta_{1}\right)g_{t} \\ v_{t}=\beta_{2}m_{t-1}+\left(1-\beta_{2}\right)g_{_{t}}^{2} \end{matrix}$ | (7-10) |
| --- | --- |

Where *g_t_* is the computed gradient at time *t_i_*, *m_t_* and *v_t_* represent first and second moment estimates of the gradient.

1. **Bias Correction:**

| $\begin{matrix} \hat{m}_{t}=\frac{m_{t}}{1-\beta_{1}^{t}} \\ \hat{v}_{t}=\frac{v_{t}}{1-\beta_{1}^{t}} \end{matrix}$ | (7-11) |
| --- | --- |

1. **Parameter Update Rule**

| $w^{(l)}=w^{(l)}-\eta\frac{\hat{m}_{t}}{\sqrt{\hat{v}_{t}}+\varepsilon}$ | (7-12) |
| --- | --- |

where *η* is the learning rate and ϵ prevents division by zero.

Continual learning follows the principles of gradient-based optimization to iteratively update the neural network weights. For each dataset, the model performs the forward propagation calculations.

Principles. S8 Continual Learning

Assume we have *K* datasets $\left\{D^{(1)},D^{(1)},\cdots,D^{(K)}\right\}$. Denote by $\theta^{(i)}$the network parameters at stage *i*, and let be the *i*_-th_ dataset.

**8.1 Parameter Initialization**

| $\theta_{0}^{(1)}=\theta_{init},\theta_{0}^{(i)}=\theta_{T_{i-1}}^{(i-1)},i=2,\cdots,K$ | (8-1) |
| --- | --- |

where *θ_init_* is a random or pre-trained initialization, and *T_i−1_* is the iteration at which training of stage *i−1* stops.

**8.2 Stage-wise Loss Function**

For stage *i*, we employ the mean squared error:

| $L^{(i)}(\theta)=\frac{1}{N_{i}}\sum_{j=1}^{N_{i}} \left\Vert f(x_{j}^{(i)};\theta)-y_{j}^{(i)}\right\Vert_{2}^{2}$ | (8-2) |
| --- | --- |

where *f(x;θ)* denotes the BPNN mapping.

- 1. **Gradient-Based Update**

At each iteration *t*, take Adam Optimizer.

- 1. **Dynamic Learning-Rate Scheduling and Early Stopping**

A ReduceLROnPlateau callback halves *η* if the validation loss does not improve over a preset patience, while EarlyStopping halts training and restores *θ_t_* when no significant decrease in validation loss is observed within a larger patience window.

Principles. S9 NSGA-II-PMCP

The Non-dominated Sorting Genetic Algorithm II (NSGA-II) is a population-based metaheuristic designed to approximate the Pareto front in multi-objective optimization problems. The algorithm employs the following key mechanisms:

**9.1 Non-dominated Sorting**

Solutions are ranked into hierarchical fronts based on Pareto dominance. A solution x_1_ dominates x_2_ if:

| $\left\{\begin{matrix} f_{i}(x_{1})\leq f_{i}(x_{1}) \\ \exists j\in\{1,2,\cdots,m\}:f_{j}(x_{1})\leq f_{j}(x_{1}) \end{matrix}\right.\forall i\in\{1,2,\cdots,m\}$ | (9-1) |
| --- | --- |

**9.2 Crowding Distance**

Diversity preservation is achieved by calculating the crowding distance metric:

| $CD(X)=\sum_{i=1}^{m} \frac{f_{i}(X_{next})-f_{i}(X_{prev})}{f_{i}^{\max}-f_{i}^{\min}}$ | (9-2) |
| --- | --- |

Where *Xnext* and *Xprev* denote adjacent solutions in the objective space.

**9.3 Elitist Selection**

A combined population P UQ (parent size N=300) undergoes tournament selection favoring solutions in higher fronts and larger crowding distances.

**9.4 Genetic Operators**

Simulated Binary Crossover (SBX):

| $c_{1}=0.5[(1+\beta)p_{1}+(1-\beta)p_{2}],\beta\sim\eta_{sbx}$ | (9-3) |
| --- | --- |

Polynomial Mutation:

| $x'=x+\delta,\delta\sim Bernoulli(p_{m} )$ | (9-4) |
| --- | --- |

Where $\eta_{sbx}$ controls crossover distribution, and$p_{m}$is the mutation probability.

**9.5 Dynamic Partition Monitoring**

(1) **Grid Partitioning**:

The joint objective space of *UTS* (maximized) and *K_t_* (minimized) is divided into non-uniform 5×5 grids:

**(2) Coverage Metric:**
For each generation, non-dominated solutions are mapped to grid cells:

| $C_{ij}=\sum_{k=1}^{N} \prod(UTS_{k}\in\varsigma_{UTS}^{i}\wedge Kt_{k}\in\varsigma_{Kt}^{i})$ | (9-5) |
| --- | --- |

Where N is the population size.

**(3) Restart Trigger**:
A restart is activated if any grid remains unvisited after *T* generations:

| $\exists(i,j)\in\left\{1,\ldots,5\right\}^{2}:\sum_{t=1}^{T} C_{ij}^{t}=0$ | (9-6) |
| --- | --- |

**9.6 Chaotic perturbation**

**(1) Chaotic Sequence**:
Perturbations are generated using the Logistic map:

| $\xi_{n+1}=\mu\xi_{n}(1-\xi_{n}),\mu=3.9,\xi_{0}\sim\mu(0,1)$ | (9-7) |
| --- | --- |

(2) **Population Perturbation**:

The worst 5% of individuals (*x_worst_*) are mutated:

| $x'=x_{worst}+\eta(\xi-0.5)\circ(x^{U}-x^{L})$ | (9-8) |
| --- | --- |

Where $\eta=0.05$ scales the perturbation, ∘ denotes element-wise multiplication, and *x^U^*, *x^L^* are variable bounds.

**(3) Elite-Guided Diversification:**
Perturbation bases are sampled from an elite archive $\varepsilon$ (capacity=100):

| $x_{base}=\varepsilon_{\left\vert\xi\left\vert\varepsilon\right\vert\right\vert}$ | (9-9) |
| --- | --- |

**9.7 Hypervolume (HV) and Fitness**

**(1) Hypervolume (HV) Indicator**

The hypervolume metric quantifies the volume of the objective space dominated by the Pareto front relative to a reference point $\overline{r}$:

| $HV=Volume(\cup_{f\in p} [f_{1} ,r_{1} ]\times[f_{2} ,r_{2} ])$ | (9-9) |
| --- | --- |

Where *P* is the obtained Pareto front; f = (*f_1_*, *f_2_*) are the objective values (*UTS*, *K_t_*); r is the anti-optimal reference point.

**(2) Composite Fitness Metric**

The normalized fitness score evaluates convergence and balance between objectives:

|  | (9-10) |
| --- | --- |

Code and model acquisition

We have made the code and trained machine learning models publicly available at: <https://github.com/befast1221280213218/Matrix-interface-reinforce-inverse-optimization-and-tailorment>.

Reference

[1] I. Alfonso, O. Navarro, J. Vargas, A. Beltrán, C. Aguilar, G. González, I. Figueroa, Compos. Struct. 2015, 127, 420-425.

[2] D. Stathokostopoulos, D. Chaliampalias, E. Stefanaki, G. Polymeris, E. Pavlidou, K. Chrissafis, E. Hatzikraniotis, K. Paraskevopoulos, G. Vourlias, Appl. Surf. Sci. 2013, 285, 417-424.

[3] Y. Li, T. Ma, Y. Ren, T. Liu, X. Zou, Mater. Res. Express 2020, 7(3), 036533.

[4] H. Chen, Y. Wu, L. Zhan, Z. Zhang, J. Zhang, Journal of Northwest Normal University(Natural Science) 2019, 55(5), 29-32.

[5] <https://www.nanorh.com/product/aluminium-silicide-nanoparticles/>.

[6] J. Cao, C.F. Li, Y. Wang, S. Fang, Appl. Mech. Mater. 2014, 529, 62-70.

[7] S. Li, Q. Su, X. Wang, Y. Wu, X. Zhao, Y. Chang, Modell. Simul. Mater. Sci. Eng. 2022, 30(8), 085012.

[8] A. Ravanan, I. Palanivel, B. Kulendran, Chiang Mai J. Sci 2022, 49, 1217-1232.

[9] A. Ravanan, I. Palanivel, B. Kulendran, Trans. Indian Inst. Met. 2024, 77(9), 2555-2563.

[10] D. Yang, F. Qiu, W. Zhao, P. Shen, H. Wang, Q. Jiang, Mater. Des. 2015, 87, 1100-1106.

[11] L.-J. Zhang, F. Qiu, J.-G. Wang, Q.-C. Jiang, Sci. Eng. Compos. Mater. 2017, 24(2), 245-251.

[12] Y.-Y. Gao, F. Qiu, R. Geng, W.-X. Zhao, D.-L. Yang, R. Zuo, B.-X. Dong, X. Han, Q.-C. Jiang, Mater. Charact. 2018, 141, 156-162.

[13] W. Kim, C. Chung, D. Ma, S. Hong, H. Kim, Scr. Mater. 2003, 49(4), 333-338.

[14] J. Zhou, M. Hayden, X. Gao, Proc. Inst. Mech. Eng. C J. Mec. Eng. Sci. 2013, 227(5), 883-895.

[15] X. Gao, X. Zhang, A. Li, L. Geng, J. Compos. Mater. 2020, 54(15), 1977-1985.

[16] S. Qu, T. Siegmund, Y. Huang, P. Wu, F. Zhang, K. Hwang, Compos. Sci. Technol. 2005, 65(7-8), 1244-1253.

[17] L. Weng, Y. Shen, T. Fan, J. Xu, Jom 2015, 67(7), 1499-1504.

[18] X. Gao, X. Zhang, L. Geng, Mater. Sci. Eng. A 2019, 740, 353-362.

[19] K. Liu, Y. Su, X. Wang, Y. Cai, H. Cao, Q. Ouyang, D. Zhang, Compos. Part B Eng. 2023, 248, 110350.

[20] M. Al-Furjan, M.H. Hajmohammad, X. Shen, D.K. Rajak, R. Kolahchi, J. Alloys Compd. 2021, 886, 161261.

[21] J. Zhang, M. Qian, X. Zhang, A. Li, L. Geng, Compos. Part A Appl. Sci. Manuf. 2025, 194, 108926.

[22] M. Li, S. Ghosh, O. Richmond, Acta Mater. 1999, 47(12), 3515-3532.

[23] G. Liu, T. Chen, Z. Wang, Mater. Sci. Eng. A 2021, 817, 141413.

[24] I. Tirtom, M. Güden, H. Yıldız, Comp. Mater. Sci. 2008, 42(4), 570-578.

[25] E. Maire, D. Wilkinson, J. Embury, R. Fougeres, Acta Mater. 1997, 45(12), 5261-5274.

[26] S. Ho, A. Saigal, Acta Metall. Mater. 1994, 42(10), 3253-3262.

[27] M. Wang, B. Song, Q. Wei, Y. Shi, J. Alloys Compd. 2019, 810, 151926.

[28] A. Knowles, X. Jiang, M. Galano, F. Audebert, J. Alloys Compd. 2014, 615, S401-S405.

[29] Y. Peng, H. Zhao, J. Ye, M. Yuan, L. Tian, Z. Li, Y. Liu, J.a. Chen, Compos. Struct. 2023, 305, 116545.

[30] X. Gao, et al., Materials 2024, 17(3), 597.

[31] X. Gao, et al., Appl. Compos. Mater. 2024, 31(4), 1457-1473.

[32] S. Zhu, D. Wang, B. Xiao, Z. Ma, Compos. Part B Eng. 2022, 236, 109851.

[33] X. Wang, X. Wang, K. Liu, H. Cao, Y. Su, D. Zhang, Q. Ouyang, Compos. Part B Eng. 2023, 259, 110708.

[34] X. Wang, S. Wang, X. Wang, Y. Su, Z. Yue, H. Cao, D. Zhang, Q. Ouyang, Mater. Sci. Eng. A 2024, 892, 145999.

[35] Z. Lin, Y. Su, C. Qiu, J. Yang, X. Chai, X. Liu, Q. Ouyang, D. Zhang, Scr. Mater. 2023, 224, 115135.

[36] Y. Su, Q. Ouyang, W. Zhang, Z. Li, Q. Guo, G. Fan, D. Zhang, Mater. Sci. Eng. A 2014, 597, 359-369.

[37] M. Kutzhanov, A. Matveev, D. Kvashnin, S. Corthay, A. Kvashnin, A. Konopatsky, A. Bondarev, N. Arkharova, D. Shtansky, Mater. Sci. Eng. A 2021, 824, 141817.

[38] T. Gao, D. Wang, X. Du, D. Li, X. Liu, J. Alloys Compd. 2016, 685, 91-96.

[39] Y. Hong, J. Liu, Y. Wu, J. Alloys Compd. 2023, 949, 169895.

[40] S. Duan, D. Zhu, J. Dong, X. Lu, D. Ding, W. Zhou, F. Luo, J. Alloys Compd. 2019, 790, 58-69.

[41] Z. Zhong, J. An, D. Wu, N. Gao, L. Liu, Z. Wang, F. Meng, X. Zhou, T. Fan, Int. J. Mech. Sci. 2024, 281, 109550.
